# Widespread distribution of bacteria containing PETases with a functional motif across global oceans

**DOI:** 10.1093/ismejo/wraf121

**Published:** 2025-06-10

**Authors:** Intikhab Alam, Ramona Marasco, Afaque A Momin, Nojood Aalismail, Elisa Laiolo, Cecilia Martin, Isabel Sanz-Sáez, Begoña Baltá Foix, Elisabet L Sá, Allan Kamau, Francisco J Guzmán-Vega, Tahira Jamil, Silvia G Acinas, Josep M Gasol, Takashi Gojobori, Susana Agusti, Daniele Daffonchio, Stefan T Arold, Carlos M Duarte

**Affiliations:** Center of Excellence for Smart Health, Computer, Electrical and Mathematical Science and Engineering Division, King Abdullah University of Science and Technology, Thuwal 23955-6900, Saudi Arabia; Biological and Environmental Science and Engineering Division, King Abdullah University of Science and Technology, Thuwal 23955-6900, Saudi Arabia; Center of Excellence for Smart Health, Biological and Environmental Science and Engineering Division, King Abdullah University of Science and Technology, Thuwal 23955-6900, Saudi Arabia; Marine Science Program, Biological and Environmental Science and Engineering Division, King Abdullah University of Science and Technology, Thuwal 23955-6900, Saudi Arabia; Marine Science Program, Biological and Environmental Science and Engineering Division, King Abdullah University of Science and Technology, Thuwal 23955-6900, Saudi Arabia; Marine Science Program, Biological and Environmental Science and Engineering Division, King Abdullah University of Science and Technology, Thuwal 23955-6900, Saudi Arabia; Departament de Biologia Marina i Oceanografia, Institut de Ciències del Mar—Consejo Superior de Investigaciones Científicas, Pg. Marítim de la Barceloneta 37-49, Barcelona 08003, Spain; Departament de Biologia Marina i Oceanografia, Institut de Ciències del Mar—Consejo Superior de Investigaciones Científicas, Pg. Marítim de la Barceloneta 37-49, Barcelona 08003, Spain; Departament de Biologia Marina i Oceanografia, Institut de Ciències del Mar—Consejo Superior de Investigaciones Científicas, Pg. Marítim de la Barceloneta 37-49, Barcelona 08003, Spain; Center of Excellence for Smart Health, Computer, Electrical and Mathematical Science and Engineering Division, King Abdullah University of Science and Technology, Thuwal 23955-6900, Saudi Arabia; Center of Excellence for Smart Health, Biological and Environmental Science and Engineering Division, King Abdullah University of Science and Technology, Thuwal 23955-6900, Saudi Arabia; Center of Excellence for Smart Health, Computer, Electrical and Mathematical Science and Engineering Division, King Abdullah University of Science and Technology, Thuwal 23955-6900, Saudi Arabia; Departament de Biologia Marina i Oceanografia, Institut de Ciències del Mar—Consejo Superior de Investigaciones Científicas, Pg. Marítim de la Barceloneta 37-49, Barcelona 08003, Spain; Departament de Biologia Marina i Oceanografia, Institut de Ciències del Mar—Consejo Superior de Investigaciones Científicas, Pg. Marítim de la Barceloneta 37-49, Barcelona 08003, Spain; Biological and Environmental Science and Engineering Division, King Abdullah University of Science and Technology, Thuwal 23955-6900, Saudi Arabia; Department of Life Sciences, National Cheng Kung University, No. 1 University Road, Tainan City 701401, Taiwan (R.O.C.); Marine Science Program, Biological and Environmental Science and Engineering Division, King Abdullah University of Science and Technology, Thuwal 23955-6900, Saudi Arabia; Biological and Environmental Science and Engineering Division, King Abdullah University of Science and Technology, Thuwal 23955-6900, Saudi Arabia; Department of Agriculture, Forestry and Food Sciences, University of Turin, Grugliasco, Turin 10095, Italy; Center of Excellence for Smart Health, Biological and Environmental Science and Engineering Division, King Abdullah University of Science and Technology, Thuwal 23955-6900, Saudi Arabia; Marine Science Program, Biological and Environmental Science and Engineering Division, King Abdullah University of Science and Technology, Thuwal 23955-6900, Saudi Arabia

**Keywords:** plastic pollution, marine microbiome, enzymatic activity, functional motifs, PET degradation, microbial diversity, functional adaptation, biodegradation

## Abstract

Accumulating evidence indicates that microorganisms respond to the ubiquitous plastic pollution by evolving plastic-degrading enzymes. However, the functional diversity of these enzymes and their distribution across the ocean, including the deep sea, remain poorly understood. By integrating bioinformatics and artificial intelligence-based structure prediction, we developed a structure- and function-informed algorithm to computationally distinguish functional polyethylene terephthalate-degrading enzymes (PETases) from variants lacking PETase activity (pseudo-PETase), either due to alternative substrate specificity or pseudogene origin. Through *in vitro* functional screening and *in vivo* microcosm experiments, we verified that this algorithm identified a high-confidence, searchable sequence motif for functional PETases capable of degrading PET. Metagenomic analysis of 415 ocean samples revealed 23 PETase variants, detected in nearly 80% of the samples. These PETases mainly occur between 1,000 and 2,000 m deep and at the surface in regions with high plastic pollution. Metatranscriptomic analysis further identified PETase variants that were actively transcribed by marine microorganisms. In contrast to their terrestrial counterparts—where PETases are taxonomically diverse—those in marine ecosystems were predominantly encoded and transcribed by members of the *Pseudomonadales* order. Our study underscores the widespread distribution of PETase-containing bacteria across carbon-limited marine ecosystems, identifying and distinguishing the PETase motif that underpins the functionality of these specialized cutinases.

## Introduction

Due to exponential growth in plastic production and poor waste management practices, over 150 million tons of plastic waste have been delivered to the ocean since 1950 [1, 2], where it harms marine life, from small invertebrates to whales [3, 4]. Plastic polymers, such as polyethylene terephthalate (PET), are derived from oil hydrocarbons and are designed to be durable in the environment [5, 6], which makes them mainly resistant to microbial degradation. However, a newly evolved plastic-degrading enzyme, PET hydrolase (PETase), was discovered in 2016 in the betaproteobacterium *Ideonella sakaiensis* isolated from a bottle-recycling facility in Japan [7]. This hydrolase—which retains the ancestral α/β-hydrolase fold but exhibits a more open active-site cleft than homologous cutinases—has presumably evolved spontaneously following massive exposure to PET-rich waste [8]. This PETase catalyzes the hydrolysis of PET to monomeric bis(2-hydroxyethyl) terephthalate (BHET) and then to mono(2-hydroxyethyl) terephthalate (MHET), which is ultimately broken down into non-hazardous monomers, terephthalate (TPA) and ethylene glycol by an additional enzyme, the MHET hydrolase (MHETase). *I. sakaiensis* possesses both enzymes (PETase and MHETase) that likely act synergistically to depolymerize PET and use it as a primary carbon and energy source [7]. However, *I. sakaiensis* PETase (*Is*PETase) showed low degradation efficiency for crystalline PET [9–12], suggesting that this specific variant is not yet fully optimized for this anthropogenic substrate [13]. Indeed, it has been shown that introducing just five key point mutations significantly enhances the activity of *Is*PETase by several orders of magnitude [9], while the selection of two preferred mutations via comparison with homologous protein sequences led to enzymatic properties that outperformed most of the other variants reported so far [14]. These findings, among others, support the idea that given sufficient time and continued exposure to PET, microbial populations may naturally evolve more efficient PET-degrading enzymes [13, 15]. Such a process is also influenced by environmental physicochemical factors, such as temperature and pH, which contribute to shaping the intrinsic properties of the enzymes (including stability and specificity), as well as their adaptation and overall performance [16, 17].

Despite being an emerging pollutant in our oceans, plastic also creates unique ecological niches by physically supporting microbial colonization, fostering the development of characteristic microbial communities dominated by bacteria, fungi, and diatoms [18–23]. These microorganisms colonize plastic particles as novel habitats and, in some cases, actively contribute to their degradation, using plastic-derived compounds as “food” [18, 20]. However, whether these microbial communities have spontaneously evolved plastic-degrading activities due to exposure to such synthetic polymers is poorly understood. Considering that the first PETase was detected in a bacterium associated with a PET bottle recycling facility [7], it is plausible that also the accumulation of PET in marine environments promotes a similar process by driving spontaneous modifications of microbial alpha/beta hydrolases into PETase enzyme variants (e.g. mutations of cutinases/lipases) [8]. This hypothesis is supported by the fact that the vast population of prokaryotes in the global ocean—estimated to be 10^29^ cells distributed among 10^10^ different operational taxonomic units [24]—would provide enough population size, genetic diversity, and growth rate for gene mutations [25] that could create novel functional PETases [3]. Therefore, given the generally oligotrophic nature of ocean environments [26], microorganisms encoding for a functional PETase would have a selective advantage by accessing an additional carbon and energy source [27, 28].

A recent global assessment detected the presence of PET hydrolase genes in 31 of the 108 marine surface water samples analysed (<2 m depth) [29]. Additionally, 1598 sequences homologous to *Is*PETase were detected from marine ecosystems across the entire water column, including the deep sea [30]. However, given that plastic pollution in our oceans is a relatively recent phenomenon, it is reasonable to expect the presence of “intermediate” PETase forms—enzymes that show structural features indicative of adaptation to PET but lack the catalytic efficiency needed for effective PET degradation under environmental conditions. Additionally, many hydrolases with structural and sequence similarity to *bona fide* PETases may instead be adapted to other substrates. Therefore, to comprehensively assess the diversity of PET-degrading enzymes within bacterial communities across the oceans, we need to accurately distinguish genes that encode functional PETases from those that produce structurally similar enzymes (here defined as pseudo-PETase) without significant catalytic PETase activity. The accurate identification of functional PETase variants at a large scale would provide more confident insight into the interplay between microbial functional evolution (i.e. mutational refinement) and environmental selective pressure (i.e. presence of PET as a substrate) in ocean microbial communities. More specifically, it could help understand the competitive advantage that efficient PET-degrading variants confer to microorganisms in exploring novel ecological niches where PET may serve as a primary carbon and energy source [31]. To this end, we developed a computational approach for PETase identification based on protein motifs that can efficiently predict a functional PETase from sequence alone and assign different levels of PETase efficacy to these sequences. We applied this method to identify PETase candidates in publicly available metagenome datasets from the *Tara* Oceans [32] and Malaspina Expedition [33] samples. Our functional predictions were further verified experimentally by assessing PETase activity (i) *in vitro* using heterologously expressed enzymes and (ii) *in vivo* through microcosm experiments with marine bacterial isolates. We also evaluated the expression of predicted functional PETase variants in metatranscriptomic datasets (i.e. *metaT* from *Tara* Ocean [34]) to determine whether such genes are actively transcribed and likely translated, as transcription and translation are tightly coupled in prokaryotic systems [35]. Our results showed that PETases are limited compared to pseudo-PETase genes that likely serve functions not related to PET degradation. Yet, PETases are primarily encoded and transcribed by members of the order *Pseudomonadales* and phylum *Gemmatimonadota* across the global oceans, with the highest abundance observed in bacterial communities of bathypelagic and surface waters in areas with elevated plastic concentrations. These findings support that encoding PETases can be advantageous for survival and proliferation in PET-exposed environments [36] and enhance our understanding of the diversity and prevalence of the PET biodegradation capacity in marine microorganisms.

## Materials and methods

### Data collection and KAUST Metagenomic Analyses Platform-based annotation

**Figure 1:**
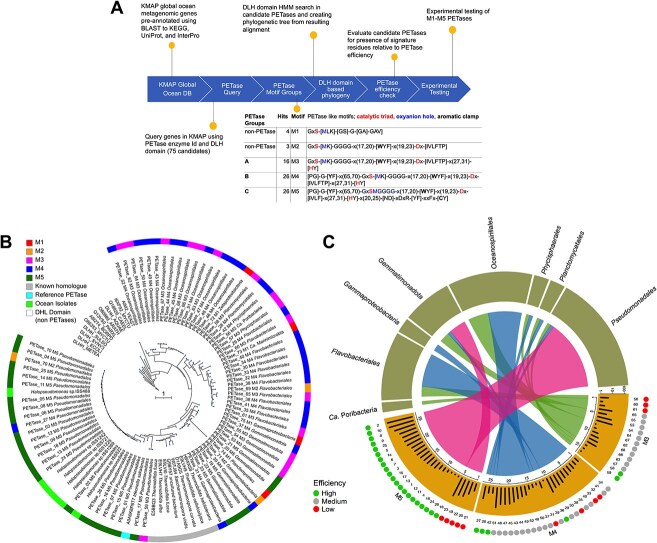
(A) Overview, phylogenetics, and efficiency analysis of candidate PETases retrieved from marine metagenomes. (B) RaxML-based phylogenetic analysis of DLH-containing PETase homologs from *Tara* oceans and Malaspina Expedition (Supplementary Data S1). The outer ring of the phylogenetic tree is colored according to reference PETase from *I. sakaiensis* (cyan), known homologs (gray), associated motifs M1–M5 (M1 in red, M2 in orange, M3 in pink, M4 in blue, and M5 in green), DLH seed sequences are in white. The scale bar represents one amino acid substitution per site. The M5 genes tested *in vitro* in this study are marked with blue stars, while the PETase_12.M5 that matches the one from isolate ISS-1242 tested *in vivo* in this study is marked with a red star. The M5 gene (PETase_02.M5), whose activity was previously shown *in vitro* [46], is marked with a black star. (C) Analysis of candidate PETases is shown for motif groups M3, M4, and M5 (lower half of the circle), presenting the potential for efficiency (outer lower circle) as green, gray, and red dots depicting high, medium, and low-efficiency categories, respectively. For potential efficiency to degrade PET, based on the evaluation of only critical residues (Supplementary Table S1) and the efficiency scoring scheme (Supplementary Data S2). The total gene abundance for each PETase in the ocean is shown in respective bar graphs with an orange background (Supplementary Data S3). Associated taxonomic information (upper half of the circle) is linked to PETase motif groups through colored ribbons (M3 is green, M4 is blue, and M5 is shown in pink).

Metagenomes publicly available from two global expeditions—the *Tara* Oceans [32] and Malaspina Expedition (Malaspina Deep [MD] and Malaspina Profile [MP]) [33, 37, 38]—were used for further analyses. The metagenomic datasets were retrieved from ENA (PRJEB40454 and PRJEB1787) and [37, 38], including 243 metagenome samples from the *Tara* Oceans and 172 metagenomes from the Malaspina Expedition. The 415 metagenomes were obtained from samples collected along the entire water column, from the surface to 4,000 m depth (Supplementary Data S1), encompassing 206 locations (Supplementary Fig. S1). The downloaded metagenomes were processed through quality control, metagenomic assembly, full-length gene prediction, and clustering of genes across samples to produce a unique set of genes, referred to as gene catalogs, which we annotate using the KAUST Metagenomic Analyses Platform (KMAP) [39]. KMAP investigates the taxonomic affiliation of every gene using Least Common Ancestor estimation through BLAST against the UniProtKB database. Briefly, a gene catalogs of non-redundant gene clusters was prepared from the metagenome datasets, where each cluster contained nucleotide sequences with ≥95% sequence identity over ≥90% of their length. We pre-processed the individual samples for quality control and validation of the pairs using bbduk (http://jgi.doe.gov/data-and-tools/bb-tools/). The metagenomic assembly was carried out using MEGAHIT version 1.2.9 [40] with default settings, except for the minimum contig size kept at 500 bp, and gene prediction was performed using Prodigal version 26.3 with the “*-c*” *option* to prevent partial genes. We performed gene clustering through the Cluster Database at High Identity with Tolerance (CD-HIT) program version 4.8.1 to obtain a unique, non-redundant set of genes across samples (95% nucleotide identity and 80% overlap of the shorter sequence). For per-sample gene abundance estimates, read mapping was performed against gene catalogs using bbmap (http://jgi.doe.gov/data-and-tools/bb-tools/) options ambig = toss idfilter = 0.9 tossbrokenreads to obtain fragments per kilobase per million mapped reads (FPKM) values. *Tara* Oceans’ microbial gene catalog was already available, and it had 40 million genes [39]. Two gene catalogs from the Malaspina Expedition, i.e. MP and MD gene catalogs, produced a non-redundant set of 32 and 11 million genes, respectively. Putative PETase genes were identified from KMAP annotations based on the presence of the PF01738 dienelactone hydrolase (DLH) family domain—a characteristic feature of PETases—identified using InterProScan. We established additional bioinformatic filters based on the sequence, functional, and 3D structural data derived from confirmed and functionally tested PETase enzymes [8, 41–45] (PDB accessions 4CG2, 4 EB0, 4WFI, 5XJH, 5YNS, 5ZNO, 6EQD, 6EQE, 6EQF, 6EQG, 6EQH, and 6KY5) and formulated these filters as five motifs (M1–M5) with increasing stringency (Fig. 1 and Supplementary Data S1). In addition, to determine whether PETase variants could be secreted extracellularly—an essential prerequisite for PET-degrading activity in the environment—signal peptide prediction was performed using the SignalP 6.0 web server [47]. This tool employs protein language models (LMs) to identify N-terminal signal peptides and their probable cleavage sites in each amino acid sequence.

### Phylogenetic analysis of polyethylene terephthalate hydrolase from oceans

A protein sequence set of the 75 non-redundant PETase-related genes obtained through querying KMAP was combined with the *I. sakaiensis* PETase gene (*Is*PETase) [7] and its eight homologs [29]; 12 seed sequences from the DLH domain were included as outgroup. The alignment of sequences was performed using hmmsearch version 3 with the DLH domain as a query. The resulting alignment was converted into fasta format, and a phylogenetic tree was built using the Randomized Axelerated Maximum Likelihood (RaxML) method [48] with 100 bootstraps and using model PROTGAMMAAUTO. To identify closer taxonomic affiliation at the species level, we compared each PETase protein sequence using BLAST in NCBI’s reference sequence database (RefSeq). The top 10 full-length protein sequences were downloaded and clustered using CD-HIT [49], considering a 99% identity threshold. The selected sequences were aligned, including *Is*PETase, and a phylogenetic tree was built using the Mafft alignment software web server at https://mafft.cbrc.jp/alignment/server/index.html.

### Efficiency scoring and homology modeling

A scoring system was developed to rank the candidate PETases, eight known PET hydrolases, and 12 seed sequences containing the DLH domain. This scoring was made by comparison to the PETase sequence from *Is*PETase, considering some of the key residues that are known to contribute to the activity and stability of *Is*PETase, as well as variants and mutations that have been experimentally tested and yielded either an increase or decrease in its ability to degrade PET (Supplementary Table S1 and Data S2). The scoring was then performed with the following considerations: given the difficulties in predicting how the presence of additional varying residues in the sequence with respect to *Is*PETase might affect the performance of the experimentally tested mutations as reported in the literature, the scoring was applied as a count or sum of the number of positive mutations (i.e. enhancing PETase activity) minus the number of negative mutations (i.e. inhibiting PETase activity). Additionally, a higher weight was given to the residues composing the catalytic triad and the PETase-specific disulfide bond, and a higher penalty for their absence. Based on this, PETases were binned into low, medium, and high efficiency. After several rounds of scoring different parameters, such as the number of mutations/key amino acids considered and the weights given to each residue, the ranking of the analysed sequences was robust. High-confidence structural models were produced for PETases with M3, M4, and M5 motifs with AlphaFold2 [50] to support and inform the scoring process. The minimum obtained pLDDT score was 83, with 75% of models having a score higher than 90 (average = 92). The models were produced in the Ibex computer cluster at KAUST, allocating 16 CPUs, 2 V100 GPU cards, and 128GB of RAM. The structural analysis of potential PETases is described in detail in Supplementary Result S1.

### Cloning, expression, and purification of recombinant polyethylene terephthalate hydrolase variants

Sequences for all constructs tested *in vitro* with a C-terminal 8xHis-tag were codon-optimized for expression in *Escherichia coli*, synthesized by TWIST Bioscience, and sub-cloned in the pJEx411c vector with kanamycin resistance. The recombinant protein expression constructs included a periplasm-targeting signal peptide identified by SignalP 6.0 that was cleaved off (if present in the sequence, Supplementary Table S2) to obtain only the desired PETase sequence. Protein constructs were transformed into Rosetta-Gami B (DE3) cells of the *E. coli* strain. Cells were grown at 37°C until OD_λ = 600_ reached 0.6. Cultures were induced with 0.5 mM IPTG and grown at 20°C for 18 hours. Cells were pelleted using centrifugation at 9000 rpm for 10 min at 4°C and stored at −80°C. Cell pellets were resuspended in a binding buffer (50 mM Tris–HCl, 200 mM NaCl, pH 8.0) and lysed using ultrasonication at 35% amplitude for 12 min (1 second on, 1 second off). The cell lysate was centrifuged at 27000 rpm for 20 minutes, and the supernatant was collected and applied to the His-Trap affinity column (Cytiva) using a binding buffer. The column was washed with 3 column volumes of binding buffer, and the proteins were eluted using 50 mM Tris–HCl, 200 mM NaCl, 500 mM imidazole, pH 8.0. Further purification was performed with the MonoQ ion-exchange column (Cytiva) using a low salt buffer (50 mM Tris–HCl, 50 mM NaCl, pH 8.0) and a high salt buffer (50 mM Tris–HCl, 1000 mM NaCl, pH 8.0). Proteins were then applied to size exclusion chromatography using a Superdex 200 10/300 column (Cytiva) in SEC buffer (20 mM HEPES, 200 mM NaCl, pH 7.5). Purity was checked using SDS-PAGE (Supplementary Fig. S2A), and all proteins were stored at −80°C upon flash-freezing using liquid nitrogen.

### Differential scanning fluorometry and para-nitrophenol enzyme activity assay

The experiment was performed as previously described [51]. Briefly, thermal stability for all the purified proteins was assessed in 20 mM HEPES, pH 7.5, and 200 mM NaCl in the presence and absence of Ca^2+^. The scan temperature ranged from 25°C to 95°C. All proteins were tested at 10 μM, and the fluorescent dye SYPRO Orange was used at a final concentration of 5X. Purified enzymes were incubated with increasing concentrations of para-nitrophenol (pNP)-acetate (Sigma-Aldrich) in 45 mM Na_2_HPO_4_-HCl (pH 7.0), 90 mM NaCl, and 10% acetonitrile at 30°C. pNP production was monitored continuously at 405 nm as previously described [7]. pNP production without the enzyme was also calculated and subtracted from the values measured in the presence of the enzymes as a baseline. The activity was calculated using the Michaelis–Menten equation using non-linear fitting to calculate the initial catalytic rate, and data were plotted using GraphPad Prism 9.0.

### Enzymatic functional assay with polyethylene terephthalate film and polyethylene terephthalate granules

Transparent PET films with 0.1 mm thickness (300 × 300 mm) and PET granules were ordered from Sigma-Aldrich. PET films were cut into 8 mm diameter using a hole puncher and washed with MilliQ water, followed by 20% ethanol, 1% SDS, and finally with MilliQ water. Purified enzymes were incubated with the PET films in 50 mM glycine-NaOH (pH 9.0) as described [7]. The reaction was terminated by diluting the tube with 18 mM phosphate buffer (pH 2.5) and heating it to 85°C for 15 min. After the reaction, both PET films and PET granules were washed and air-dried for scanning electron microscopy (SEM) imaging, as mentioned above. The samples were then mounted on a 3.2 mm SEM sample stage with carbon tapes and coated with 8 nm platinum/palladium using a Cressington Sputter coater. SEM imaging was performed under vacuum using a Zeiss SEM. The electron beam intensity was 5 kV, and images were taken at 2500× at a 10 μM distance.

### Screening of marine *Halopseudomonas* (formerly *Pseudomonas*) strains for polyethylene terephthalate film degradation in microcosm experiments

An initial PCR-based screening of the PETase gene conducted on the marine heterotrophic bacterial culture collection (MARINHET) [52] (Supplementary Method S1) allowed the identification of six PETase-positive isolates. Among them, the ISS-721, ISS-1225, and ISS-1242 strains were selected to screen their PET degradation activity *in vivo*. The genome of the three strains was sequenced (Supplementary Method S2) and analysed (Supplementary Result S2). Average Nucleotide Identities (ANI) comparison between complete genomes from isolates and closer species revealed that the isolates belong to *H. pachastrellae* [53, 54]*,* with ISS-1242 forming a separate sub-cluster from ISS-1225 and ISS-721 (Supplementary Fig. S3). Glycerol stock of the three strains ISS-721, ISS-1225, and ISS-1242 were recovered on Zobell Marine Broth (MB) 2216 medium (HiMedia; composition: 5 g peptone, 1 g yeast extract, 0.1 g C_6_H_5_FeO_7_, 19.45 g NaCl, 5.9 g MgCl_2_, 3.24 g MgSO_4_, 1.8 g CaCl_2_, 0.55 g KCl, 0.16 g NaHCO_3_, 0.08 g KBr, 34 mg SrCl_2_, 22 mg H_3_BO_3_, 4 mg Na_2_SiO_3_, 2.4 mg NaF, 1.6 mg NH_4_NO_3_, 8 mg Na_2_HPO_4_) at 24°C for 24 h. As initial screening, the PET degradation ability was tested by using different liquid media in the presence and absence of C-sources: (i) modified MB without C (mMB, the medium contained all components except yeast extract and peptone), (ii) modified MB with only 0.01% of yeast extract (mMB + C), (iii) Red Sea water (SW), and (iv) SW with the addition of 0.05% of yeast extract and 0.25% of peptone (SW + C). Each bacterial strain was grown overnight in MB medium, and cell concentration was determined using a Thoma Chamber. An appropriate amount of culture was collected to inoculate 10^6^ bacterial cells per flask containing 300 mL of medium and six pieces of PET film. The PET films (0.1 mm thick, Sigma-Aldrich GF09063581) were cut into 96 × 24 mm (film weight, 0.3157 ± 0.0016 g), incubated in 70% ethanol for 5 min, rinsed with sterile water, and finally dried for 24 h at 50°C. Additional flasks not inoculated with bacteria and supplemented with PET films were used as controls. Flasks were incubated on a shaker (100 rpm) for 37 days at 25°C ± 1°C. The microcosms were refreshed every 10–14 days by substituting 2/3 of the volume with a fresh medium by letting the medium settle for 1 hour before taking out the solution. It allowed the introduction of fresh nutrients and energy sources, keeping enough bacterial cells in each microcosm for further generations. The procedure was done for all replicates, including the negative controls. The PET films were extracted from the flasks at different times, washed with 70% ethanol for 5 minutes and dried in a hot-air oven at 50°C for 1 h. The degradation of the PET films was evaluated in terms of weight loss and expressed as a percentage: *W_0_-W_f_ / W_0_* ✕ 100, where *W_0_* and *W_f_* are the initial and final weights of the PET films (g), respectively. Based on the initial results (Supplementary Fig. S4), further characterization of the PET degradation/deterioration dynamics mediated by strains was performed in SW by adding 0.05% yeast extract and 0.25% peptone (SW + C), following the same protocol. Two independent incubations of 85 and 64 days were performed in triplicate. In the last experiment, we also included an additional microcosm set as a control, in which we inoculated *E. coli* BL21 DE3 grown in Luria–Bertani (LB) medium. The obtained cells were inoculated in flasks filled with minimal M9 medium instead of seawater, following previously described procedures.

### Scanning electron microscopy of polyethylene terephthalate films co-cultivated with bacteria

To evaluate the ISS-1242 strain’s adhesion to the surface, the PET films were collected after 21 days of incubation. The samples were removed from the media and immediately fixed in a solution of 2.5% glutaraldehyde EM quality in 0.22 μm filtered autoclaved seawater. Post-fixation was performed in 2% osmium tetroxide for 1 hour, and dehydration was performed in 30–50–70–90–100% graded ethanol for 15 min for each step, followed by CO_2_ critical-point-drying by using EM CPD300 (Leica, Germany) dryer. Palladium coating was performed using a K575X sputter coater (Quorum Technologies, UK) with a final thickness of 5 nm. Analysis of the PET films’ surface was further conducted at 60 days; samples were washed in 1% SDS, distilled water, and then 70% ethanol and air-dried overnight before being placed on a support with carbon tape. The samples were then coated with palladium, as described above. Microscope images were collected with Teneo VS SEM (Thermo Fisher Scientific, US) at the Imaging and Characterization Core Lab of KAUST, using an electron beam intensity of 5 kV.

### High-performance liquid chromatography analysis

For both *in vivo* and *in vitro* PETase activity assessment, high-performance liquid chromatography (HPLC) analysis was performed [55]. Standards of BHET and TPA were obtained from Sigma Aldrich (Supplementary Fig. S5). The analysis of samples was performed on an Agilent 1260 HPLC (Agilent Technologies, Santa Clara, CA) equipped with a G1315A diode array detector (DAD). Each sample and standard were injected using a volume of 20 μL onto a Phenomenex Luna C18 column, 5 μm, 4.6 × 150 mm (Phenomenex, Torrance, CA) at 25°C. DAD detection at a wavelength of 240 nm was performed for each analyte. PET monomers were calculated using a standard curve for BHET. Raw data were obtained and plotted using GraphPad Prism 9.0.

### Assessment of M5-polyethylene terephthalate hydrolase abundance and distribution in ocean metagenomes

To assess the relative abundance of functional PETase variants, i.e. defined as those sequences containing the M5 motif (hereafter referred to as M5-PETase; see Results, Fig. 1 and Supplementary Data S1), we mapped the metagenomic reads from the *Tara* Oceans, MP, and MD datasets to the M5-PETase corresponding DNA sequences. Mapping was performed using BBMAP from the BBTOOLS package (https://archive.jgi.doe.gov/data-and-tools/software-tools/bbtools/) [39]. This allowed us to quantify M5-PETase abundance in terms of Fragments Per Kilobase of gene per Million mapped reads (FPKM), a metric that accounts for sequencing depth, gene length, and paired-end sequencing biases. To further control for differences in microbial community size across samples, M5-PETase FPKM values were normalized by the FPKM of the *recA* gene. We then aggregated the *recA*-normalized FPKM values for all the M5-PETase variants across each sample to assess the cumulative PETase functional potential of the total microbial community [34]. Geographical distribution and cumulative abundance of M5-PETases across oceans were visualized in maps using QGIS. Following the same approach, we assessed the presence of MHETase-like genes, i.e. mono (2-hydroxyethyl) terephthalate hydrolase, an enzyme that facilitates the degradation of PET by-product after PETase activity. We utilized KMAP annotation of the *Tara* Oceans and Malaspina Expedition (MP and MD) gene catalogs derived from KEGG BLAST to obtain the lists of genes assigned with MHETase-like KEGG KO ID (K21105).

### Assessment of M5-polyethylene terephthalate hydrolase transcription in global ocean metatranscriptomes

To explore the expression of PETase genes fulfilling the M5 motif (M5-PETase), we utilized a global ocean metatranscriptome (*metaT*) dataset [34] comprising 187 metatranscriptomic samples. We first used our M5-PETase sequence (12.M5, Supplementary Data S1) as a query against the *metaT* database at the Ocean Gene Atlas portal (https://tara-oceans.mio.osupytheas.fr) to identify related genes. The resulting gene set was tested with the M5 motif using PHI-BLAST. Expression values were obtained for each sample from the *metaT* gene profile (www.ebi.ac.uk/biostudies/files/S-BSST297/OM-RGC_v2_gene_profile_metaT.tsv.gz), showing gene transcript expression calculated as gene length-normalized insert count (MOCAT option profile), i.e. mean number of reads per base. We further applied the same normalization strategy used for the metagenomic data and normalized the relative abundance of these transcribed genes to that of *recA*. Similarly, we assessed the presence of MHETase transcripts, using as query MHETase-like sequence against the *metaT* expression table. Taxonomic affiliation of these expressed genes was available from [34].

### Correspondence between plastic concentrations and M5-polyethylene terephthalate hydrolase abundance

The plastic concentration (in g/km^2^) recorded during the ocean surface survey conducted by the Malaspina Expedition (http://scientific.expedicionmalaspina.es/#!/n/malaspina-digital/s209) and by other 24 expeditions [56] was taken into consideration to assess whether a correspondence between plastic abundance and M5-PETases genes or transcripts abundance was present. For each plastic sample, the closest metagenomes from the *Tara* Oceans and Malaspina Expedition used in this study or metatranscriptomes from *metaT* within a 5° radius (~550 km) were selected. When multiple metagenome samples were collected at the same location, they were all assigned to the same plastic sample. Multiple plastic samples could match with one metagenome sample when this was the only one located within a 5° radius of the said set. Plastic samples that did not have metagenome samples closer than a 5° radius were discarded. Since the plastic abundance data were obtained from ocean surface samples, only metagenomes and metatranscriptomes from samples not deeper than 10 m were included in the analyses. Applying these thresholds returned 816 records of association between plastic and metagenomes and 506 records of association between plastic and metatranscriptomes. For each sample, the sum of the M5-PETase variants’ abundance retrieved from metagenomes and metatranscriptomes was then reported. We examined the linear relationship of plastic concentration with M5-PETase-gene and M5-PETase-transcript abundance to assess whether samples containing plastic exhibited significantly higher M5-PETase abundance using the non-parametric Mann–Whitney test.

### Phylogenetic comparison of M5-polyethylene terephthalate hydrolase sequences from marine and terrestrial ecosystems

To compare the distribution of M5-PETases between marine and terrestrial ecosystems, we first performed a search using our 12.M5 variant (Supplementary Data S1) in the ocean gene atlas database [57] with BLAST database selection for metatranscriptomic catalog (*metaT*, prefix “OM-RGC v2”) and the phylogenetic option to include similar sequences from NCBI’s reference sequence database (RefSeq, prefix “WP”). The resulting sequences were subjected to the M5 motif. Additionally, we searched NCBI’s metagenomic proteins database using M5 Motif and PHI-BLAST (prefix “GB”). The M5-compliant sequences obtained, including *I.* sakaiensis wild-type PETase (*Is*PETase), were aligned to produce a Maximum Likelihood phylogenetic tree using PhyML (https://ngphylogeny.fr/). We also explored the Universal Protein Resource (UniProt) with DLH domain-containing protein sequences (PF01738), https://www.ebi.ac.uk/interpro/entry/pfam/PF01738/protein/UniProt/, yielding approximately 89,000 sequences. The sequences were subjected to the M5-PETase search, and those positive were assessed for taxonomic affiliation available from UniProt. To further explore the distribution of M5-PETases across diverse environments, we utilized the EBI metagenomic proteins database. The *Is*PETase sequence was used as a query to identify homologous sequences. These resulting hits were then subjected to the M5 motif detection. A heatmap was produced based on the taxonomic information and the count of M5-PETases across sample types. This analysis was also used to identify the earliest known protein sequences containing the M5-PETase motif. Metadata—such as collection date and annotation—were extracted from the respective databases to determine the earliest occurrences of DLH domain-containing protein sequences containing the M5 motif.

## Results

### Bioinformatic motifs discriminate potential functional polyethylene terephthalate hydrolase in the global oceans

We based our exploration of marine PETases on metagenome datasets established from two global ocean expeditions, the *Tara* Oceans [32] and the Malaspina Expedition [33, 37, 38], encompassing 415 samples from 206 locations across the Indian, Pacific, and Atlantic Oceans (Supplementary Fig. S1). These publicly available metagenomes were used to prepare a gene catalog of 83 million non-redundant gene clusters, where each cluster contained nucleotide sequences with ≥95% sequence identity over ≥90% of their length. The gene catalogs were annotated using the KAUST Metagenomic Analyses Platform (KMAP) [39] and screened for sequences similar to the *I. sakaiensis* PETase [7] (*Is*PETase EC 3.1.1.101)-like enzymes. Specifically, we searched for genes encoding enzymes that contain the DLH domain, a generic α/β-hydrolase fold found in over 80,000 protein sequences in the UniProtKB database. The DLH domain-containing sequences search identified 75 representative gene clusters in our metagenomic catalogs (Supplementary Data S1). Although these sequences contain a DLH domain, they may not necessarily be PETases. Following our motif ranking, we formulated filters as five motifs (M1–M5) with increasing stringency (Fig. 1A and Supplementary Fig. S6). The first motif, M1, only queries for a short and promiscuous PETase-associated motif derived from helix-4 that carries the catalytic serine. M2 improves the M1 filter by adding a search for a conserved four glycine motif (GGGG) that is required for embedding the helix-4 into the protein core and by adding two signatures, one for the aromatic clamp of PET substrates and one for the catalytic aspartic acid. Motif M3 adds the previous signatures for the entire catalytic triad (including the histidine), and M4 includes the [PG]-G-[YF] motif that defines an additional aromatic clamp and oxyanion hole upstream in the catalytic triad. M5 strictly defines the residue after the catalytic serine as a methionine (oxyanion hole) and adds a conserved DxDxR (Y) xxFxC sequence requirement. The M5 motif ensures correct pseudo-PETase packing of the final helix to the core and includes one of the two cysteines that form a stabilizing internal disulfide bond [29]. Among these motifs, M3–M5 contain the complete catalytic triad (Ser, Asp, and His residues) and define the sequence- and structure-guided minimum and maximum residue distances between individual motifs (Supplementary Figs S7 and S8). When searching for these motifs within our 75 PETase candidate genes, we identified 4, 3, 16, 29, and 23 representative sequences that contained (up to) the M1, M2, M3, M4, or M5 motif, respectively (Supplementary Data S1). Signal peptides for extracellular translocation (required for PETase to access the extracellular plastic) were detected in 22 out of 23 M5-PETase sequence variants, whereas their presence was variable in sequences with lower motif ranking (M1–M4) (Supplementary Table S2).

We further computed a RaxML–based bootstrapped phylogenetic tree [48] with the 75 PETase candidates, the *Is*PETase sequence [7], eight terrestrial homologs reported in the UniProt database [29], and 12 non-PETase DLH domain core sequences from the Pfam database as an outgroup (Fig. 1B). The resulting phylogenetic tree identified the 12 non-PETase DLH domain core sequences as a parental group for the candidate PETase genes, hereafter defined as clade I. All the other clusters represent six additional taxonomic clades that differentially contain the five motifs (Fig. 1B). Specifically, clade II is dominated by *Oceanospirialles* (M3 and M4), clades III and IV include a mixture of taxa but are dominated by *Flavobacteriales* (M2–M4), clade V contains *Gemmatimonadota* and *Gammaproteobacteria* (M1–M5), and clade VI contains previously defined homologs of *Is*PETase from land microorganisms [29]. The clade VII is formed by the original *Is*PETase sequence along with several sequences belonging to the *Pseudomonadales* order within the *Gammaproteobacteria* class; all contain the M5 motif except three sequences that were identified as M2, M3, and M4, respectively (Fig. 1B).

In addition to the motif filters, we established a theoretical PETase “efficiency score” (see Materials and methods) based on the presence of residue substitutions that were previously identified as enhancing PETase activity in engineered laboratory variants of the *Is*PETase [58] (Supplementary Table S1 and Data S2). The substitutions increase the hydrophobicity of the ligand binding site and reduce the steric hindrance around the active site to facilitate substrate accumulation near the binding cleft (Supplementary Result S1). This score is non-overlapping with the residues probed by the M1–M5 filters and is expected to reflect the efficacy, rather than capability, of using hydrophobic polymers such as PET as a substrate. By ranking the 75 PETases representatives from ocean metagenomes, we found that the highest efficacy scores (scores 9–11; Fig. 1C and Supplementary Data S2) were only achieved by sequences from *Pseudomonadales*, all but two (one M4 and one M2) of them were identified as M5 in our structure-based motif search. All the other sequences had efficacy scores lower than four and belonged to the M1–M4 motif classes, with the exception of the M5-PETases encoded by *Gemmatimonadota* bacteria (Supplementary Data S2). The latter has the back of the ligand binding site formed by glutamic acid and histidine rather than alanine/tryptophan as in *Is*-WT (A89 and W159)/*Pseudomonadales* M5, negatively affecting PETase activity [44].

### M5-polyethylene terephthalate hydrolase have polyethylene terephthalate-degrading activity *in vitro*

**Figure 2:**
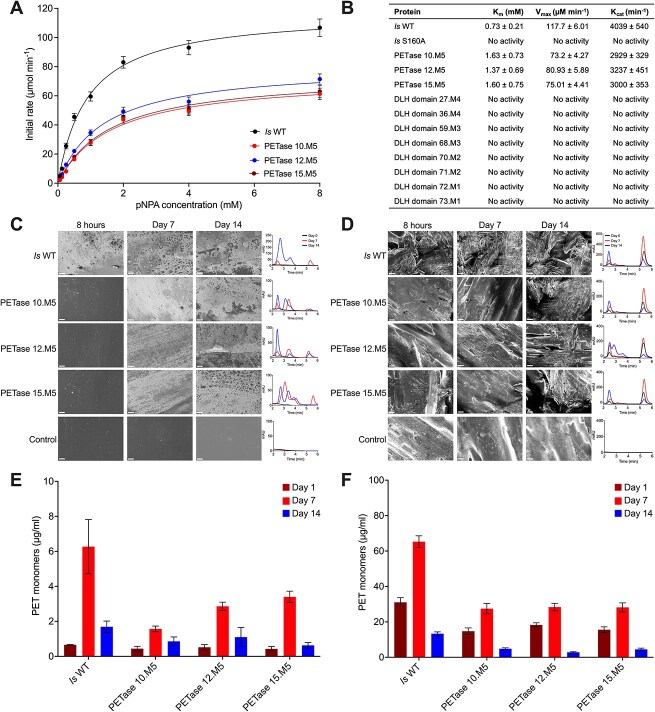
(A) p-NP esterase activity assay comparing the PETase 10.M5, PETase 12.M5, PETase 15.M5, *I. sakaiensis* WT (*is*-WT), and *I. sakaiensis* S160A (*Is*-S160A). Data are reported as mean ± SD (*n* = 3). (B) Table of catalytic parameters calculated from data presented in panel a. SEM imaging and HPLC profiles of the PET degradation assay on PET films (C) and PET granules (D) after 8 hours, on Day 7, and Day 14 of incubation. Scale bars indicate 10 μm. Quantification of the PET monomers (BHET) released after 8 hours, day 7, and day 14 of incubation on PET films (E) and PET granules (F). PET films and PET granules with their HPLC profiles in the absence of enzymes are shown in Supplementary Fig. S9. Molecular weight standards are shown in Supplementary Fig. S5.

We tested the catalytic activity of PETase motif candidates *in vitro*. We recombinantly produced 11 ocean DLH genes, with motifs ranging from M1 to M5. We also produced the *Is*PETase (*Is*-WT) and its catalytically impaired S160A mutant (*Is*-S160A) as positive and negative controls, respectively (Supplementary Data S3). All recombinant proteins were purified to >90% (Supplementary Fig. S2A). The obtained proteins of the *Is*PETase and ocean DLH variants with M5 and M4 motifs showed melting temperatures (*T_m_*) between 47–51°C (Supplementary Fig. S2B), whereas the *T_m_* of all M1–M3 enzymes range between 31°C and 42°C. The apparent molecular weight on SDS PAGE gel and AlphaFold modeling confirmed that the lower stability of the M1–M3 enzymes is explained by truncations compared to *Is*-WT (Supplementary Fig. S2A). The generic p-nitrophenol acyl esterase activity assay showed positive activity for all the M5-DLH variants tested, although their degradation efficiency was 25–50% lower than *Is*-WT (Fig. 2A and B). Degradation and structural alterations of PET substrate were also observed for both PET films and granules, on which M5 enzymes produced characteristic scratch marks that progressively deepened and widened over time (Fig. 2C and D). This enzymatic activity led to the release of PET monomers and by-products, including terephthalic acid (TPA) and mono(2-hydroxyethyl) terephthalate (MHET) (Fig. 2C–F; molecular weight standards in Supplementary Fig. S5). On the contrary, neither *Is*-S160A nor any M1–M4 enzymes showed activity in this assay (Supplementary Fig. S9). DLH variants demonstrating activity in *in vitro* functional assays all contained the M5 motif and belonged to clade VII in the phylogenetic tree (Fig. 1B), i.e. PETase_10.M5, PETase_12.M5 and PETase_15.M5 assessed in this study (Fig. 2) and PETase_02.M5 previously tested [46]. Thus, we concluded that only the M5 variants were functional PETases (hereafter M5-PETases).

### *In vivo* polyethylene terephthalate degradation by bacteria encoding a full M5-polyethylene terephthalate hydrolase gene

To validate M5-PETase activity *in vivo*, we conducted proof-of-concept experiments by assessing PET film degradation in microcosms inoculated with bacterial isolates from the marine culture collection of heterotrophic bacteria (MARINHET) [52]. Based on initial PCR-based screening, we identified six positive strains (Supplementary Table S3), of which three were selected to assess their PETase motif from genome sequencing and capabilities to degrade PET: ISS-721 isolated from 4,000 m depth in the South Atlantic (Malaspina Expedition), and ISS-1225 and ISS-1242 captured from surface water (5 m) in the North Atlantic (*Tara* Oceans). All three isolates were identified as *Pseudomonas* spp., recently reclassified as *Halopseudomonas* spp. [54] (Supplementary Fig. S3). Analysis of bacterial assembled genomes (Supplementary Method S1) revealed that only ISS-1242 encodes a complete M5-PETase motif, identical to PETase_12.M5 from *H. pachastrellae* (Fig. 1B), including both a complete catalytic fold and a secretion signal peptide (Supplementary Result S2 and Figs S10 and S11). In contrast, the other two strains possess incomplete PETase sequences, lacking key residues required for activity, and are thus classified as pseudo-PETase genes.

**Figure 3:**
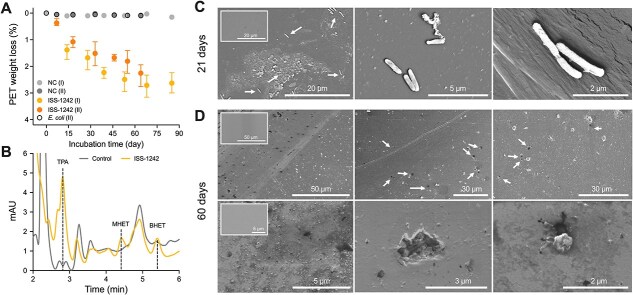
(A) Time course of PET film weight loss (%) by *Halopseudomonas* sp. ISS-1242 strain and non-inoculated control (NC) in microcosms with SW + C medium. Data are reported as an average of three replicates and their range of two distinct experiments conducted independently and indicated as (I) and (II). Microcosms inoculated with *Escherichia coli* were added as additional control with M9 + C medium during the experiment (II) (see Materials and methods). (B) HPLC spectra of culture supernatants from ISS-1242 incubated with PET in SW + C for 60 days. Control refers to the PET film incubated in the same culture medium without bacteria. The retention times of the standards (TPA, MHET, and BHET) are indicated by dashed lines. Molecular weight standards are shown in Supplementary Fig. S5. (C) SEM micrographs of PET films show the colonization of *Halopseudomonas* ISS-1242 cells at 21 days. Scale bars are indicated in each panel. The insets show PET films incubated without bacteria and used as controls. Arrowheads in the left panel indicate the bacterial cells adhering to the surface of the PET biofilm; magnification of bacterial cells in the center and right panels show contact points of the cells with the PET film surface. (D) SEM micrographs of degraded PET film at 60 days after washing out adherent cells. The insets show PET films incubated without bacteria at different magnifications (magnifications in Supplementary Fig. S13). Scale bars are indicated on each panel. Arrows indicate areas damaged extensively by the bacterial cells.

Microcosm experiments showed that PET film degradation varied among the three inoculated strains and occurred only when an additional carbon source (peptone and/or yeast extract) was supplied (Supplementary Fig. S4). This is consistent with the absence of a MHETase gene in the selected strains (Supplementary Result S2), which is required to facilitate the assimilation of PET by-products. Only strain ISS-1242—which was predicted through genome analysis to encode a functional M5-PETase—exhibited a significant reduction in PET weight compared to negative controls without bacterial inoculation (2.7% ± 0.5% at 85 days, Fig. 3A, Supplementary Fig. S12), with the highest degradation efficiency observed when using filtered/autoclaved seawater (Supplementary Fig. S4). In contrast, PET degradation by ISS-721 and ISS-1225 was negligible (< 0.5% over 85 days), with a non-significant differentiation from the negative controls (*t*-test at 64 days, ISS-721 vs control: *t* = 1.8, df = 4, *P* = .14; ISS-1225 vs control: *t* = 1.7, df = 4, *P* = .15). Such minor and inconsistent variations in PET film weight may be attributed to factors unrelated to PETase enzymatic activity, including, among others, nonspecific microbial metabolism (e.g. acidification of the medium), mechanical abrasion due to agitation, or the activity of other esterases with broader substrate specificity. Microcosms inoculated with *E. coli* showed no PET degradation activity with values comparable to negative controls (*t* = 1.7, df = 4, *P* = .16; Supplementary Fig. S12). HPLC confirmed the degradation of the PET film with the formation of mainly TPA, whereas MHET was present in low quantity (Fig. 3B). The PET-degrading strain ISS-1242 colonized the PET film surface, forming small and separate colonies without creating a homogeneous biofilm (Fig. 3C), as previously observed for other marine degraders [59]. Magnifications at single-cell resolution revealed that bacteria were anchored to the PET film (Fig. 3D), resulting in surface alteration with irregularities, erosion, deposits, cavities, and fissures (Fig. 3D) compared with control films (Supplementary Fig. S13).

The *in vivo* functional assay revealed that only bacteria encoding enzymes with the complete M5 motif—along with a signal peptide enabling extracellular secretion—exhibited significant PET-degrading activity, confirming the results of *in vitro* assays (Fig. 2) and the overall motif ranking to determine functional PETase variants proposed.

### Distribution and abundance of M5-polyethylene terephthalate hydrolase in the global ocean

**Figure 4:**
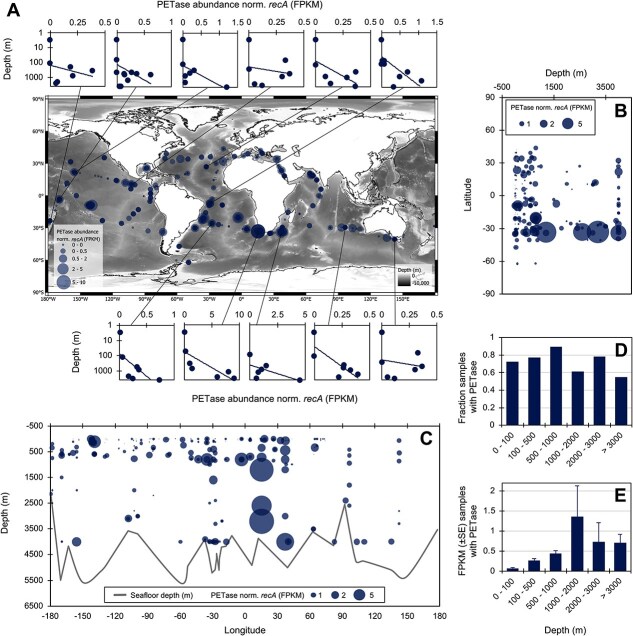
Distribution and prevalence of microbial M5-PETases across oceans. (A) Relative abundance of M5-PETases detected across 415 metagenome samples. Values are expressed as the sum of FPKM mapped reads of all detected M5-PETase variants within a sample, normalized to the FPKM of the *recA* gene within the same sample. Each point corresponds to a metagenome sample. The plots surrounding the map show the distribution of PETase abundance, normalized per *recA* abundance, across depth (m) in 11 profiles collected during the Malaspina expedition. (B and C) distribution of M5-PETase normalized per *recA* gene abundance along with depth and latitude, and along with depth and longitude. The gray line in panel C shows the seafloor depth (m). (D) Fraction of samples where M5-PETase was detected over the total number of samples at each of 6 depth categories, i.e. 0–100 m, 100–500 m, 500–1,000 m, 1,000–2,000 m, 2,000–3,000 m, and  > 3,000 m). (E) Mean (±SE) of M5-PETase abundance, normalized per *recA* gene abundance, at the same six depth layers, including only samples where M5-PETases were detected. M5-PETase normalized abundance and prevalence across metagenome samples from different filter size ranges are reported in Supplementary Fig. S16.

We analysed the distribution patterns and prevalence of M5-PETase variants across 415 marine metagenomes. To ensure comparability across samples and account for variations in microbial load, the relative abundance of M5-PETase genes was calculated as FPKM and normalized against the FPKM of the single-copy housekeeping gene *recA* (Supplementary Data S4). The M5-PETase variants were affiliated with the order *Pseudomonadales* (18 variants) and the phylum *Gemmatimonadota* (5 variants) (Supplementary Data S1) and were detected in 323 samples (Supplementary Data S4). The combination of these variants per sample defined such a differential distribution across geographic locations and along depth (mapped in Fig. 4A), with a maximum toward the ocean interior at the base of the permanent thermocline (1,000–2,000 m depth, Fig. 4B and C). At 11 sampling locations covering multiple depths, the cumulative abundance of M5-PETase variants increased significantly with depth (mixed-effect model: slope for depth = 0.4, *P* = .0032; Fig. 4A). In the upper ocean (<200 m), the abundance of M5-PETase variants was highest near South America, South Africa, India, French Polynesia, and the Red Sea, while in deeper waters their prevalence peaked between the Atlantic and Indian Oceans at depths of 200–1,000 m and  >1,000 m (Supplementary Fig. S14). This distribution was primarily associated with the presence of M5-PETases encoded by *Gemmatimonadota* and *Pseudomonadales* in the first portion of the mesopelagic ocean (100–1,000 m) and at depths below 2,000 m, respectively, while both taxonomic groups contribute to the abundance of these enzymes between 1,000 and 2,000 m (Supplementary Fig. S15). Whereas a unique bacterium does not necessarily encode both PETase and MHETase enzymes (as also shown in our isolates), MHETase genes were detected in 411 samples (Supplementary Fig. S17 and Data S5), of which 324 also harbored M5-PETase genes. This suggests that although the full PET degradation pathway may be distributed across different taxa, the metabolic potential for PET breakdown is present in co-occurring microbial communities, i.e. the same metagenome sample.

When comparing M5-PETase abundance with plastic concentration, a significant relation was detected (*n* = 816, *P* = 2 × 10^−16^, *R*^2^ = 0.09; Supplementary Fig. S18 and Data S6A). This relationship was further confirmed by a 10-fold higher M5-PETase abundance associated with samples where plastic was detected (Mann–Whitney test: *P* = 10 × 10^−13^, mean ± SE M5-PETase abundance: absence of plastic = 0.0021 ± 0.0003 FPKM [*n* = 283] vs presence of plastic = 0.028 ± 0.004 FPKM [*n* = 533]). Some regions showing a high abundance of M5-PETases, such as Southern Africa and the eastern coast of South America (South Atlantic Gyre), coincide with areas previously identified as microplastic accumulation hotspots [54–56]. Besides this relationship, we also searched for publicly available marine metagenomic datasets collected prior to the widespread production of plastic to investigate whether the emergence of M5-PETase variants may have coincided with the rise in global plastic pollution. Although no metagenomic datasets predating the 1990s were available, we identified M5-PETase sequences in the oldest accessible marine metagenomes from 1990 (Supplementary Data S7). The identified amino acid sequence originates from *Moraxella* sp. TA144, a bacterium isolated from Antarctic seawater [60]. This enzyme, annotated in UniProt as a triacylglycerol lipase (entry P19833), was recently reported to degrade highly crystalline synthetic polymers [61]. While this evidence does not establish when M5-PETases first evolved, it suggests that homologous enzymes with PET-degrading potential were already present in ocean microbial communities before the functional characterization of *I. sakaiensis* in 2016 [7]. This finding supports the hypothesis that marine bacteria may have independently repurposed ancestral hydrolases for PET degradation to exploit novel carbon and energy sources, i.e. plastic, accumulating in marine environments.

### Do marine microbial communities transcribe M5-polyethylene terephthalate hydrolase genes?

In addition to demonstrating the functionality of three M5-PETase variants *in vitro* (Fig. 2) and confirming the activity of one variant *in vivo* as for ISS-1242 (Fig. 3), we investigated whether and to what extent these genes are actively transcribed in marine microbial communities. Since transcription represents the first step of gene expression, its detection serves as a proxy for assessing the potential functionality and ecological relevance of M5-PETases in marine ecosystems. Transcriptional activity from metatranscriptomic data (*metaT* dataset) supports that M5-PETases are not only present in oceanic microbial communities as silent genes but are also actively transcribed (Supplementary Fig. S19). Specifically, six M5-PETase gene variants—including the PETase_12.M5 encoded by *Pseudomonas* strain ISS-1242—were found in 86 samples out of the 187 included in the *metaT* dataset, with a range of 1 to 4 (median = 1) variants per sample (Supplementary Data S8A). In addition, such variants were transcribed in 35 other samples from the *metaT* dataset beyond those represented in the *Tara* Oceans metagenomic gene catalog [33]. Interestingly, we found transcripts for MHETases in 163 ocean samples (Supplementary Data S8B), of which 113 samples also showed transcriptional signatures for M5-PETase, supporting the existence of a metabolic *continuum* for PET degradation at the microbial community level throughout the seawater column.

Taxonomic analysis further confirmed that transcribed PETase genes primarily originated from bacteria of the *Pseudomonadales* order, including genera such as *Pseudomonas* and members of the family *Moraxellaceae*. We also detected M5-PETases transcribed in samples from the Antarctic by members of the *Oceanospirillales* order, such as *Oleispira antarctica*. If we consider the depth profiles explored by metatranscriptomic datasets (no deeper than 800 m), it is evident that transcription of M5-PETases was mainly detected at the surface, with the exception of samples in the Pacific Ocean (Supplementary Fig. S19). Yet, when comparing M5-PETase transcript abundance with plastic concentration, a significant relation was detected (*n* = 506, *P* = 1 × 10^−5^, *R*^2^ = 0.04; Supplementary Fig. S20 and Data S6B). This relationship was further confirmed by a 10-fold higher M5-PETase transcripts abundance associated with samples where plastic was detected (Mann–Whitney test: *P* = 10 × 10^−13^, mean ± SE PETase transcripts abundance: absence of plastic = 0.002 ± 0.001 [*n* = 192] vs presence of plastic = 0.02 ± 0.005 [*n* = 314]).

### Are ocean M5-polyethylene terephthalate hydrolase different from those encoded by terrestrial microorganisms?

The metagenomic and metatranscriptomic analyses conducted on datasets from the *Tara* Ocean and Malaspina Expedition revealed that M5-PETase variants are primarily affiliated with the *Pseudomonadales* order (Fig. 1B). We thus questioned whether this taxonomic pattern represents a distinct signature of ocean-derived PETase variants or reflects a broader trend observed across diverse ecosystems, including terrestrial environments. To investigate this question, we retrieved and analysed PETase sequences matching the M5 motif across multiple publicly available repositories and databases, ensuring coverage across a wide range of environmental settings. Phylogenetic analysis revealed a stark difference in the distribution of M5-PETases between terrestrial and oceanic ecosystems (Supplementary Figs S21 and S22). While *Pseudomonadales* (i.e. *Pseudomonas* spp. and *Halopseudomonas* spp.) dominate the M5-PETase representation in marine environments, terrestrial ecosystems exhibit a broader taxonomic diversity, with *Actinobacterota* (among others, *Streptomyces*, *Actinoplanes*, *Actinokineospora*, *Saccharothrix*, *Nocardiopsis*, *Thermobifida*, *Actinomadura*), *Betaproteobacteria* (*Piscinibacter* and *Roseateles*), and *Gammaproteobacteria* (*Pseudomonas*, *Halopseudomonas*, *Vibrio*, and *Ketobacter*) encoding such gene variants (Supplementary Data S9). Although taxonomic analysis supports a distinct divergence in M5-PETase between ocean and land environments, this taxonomic confinement is associated with the stringent motif criteria. When expanding the analysis to include DLH genes with motifs M1–M4, a broader taxonomic distribution also emerged in marine environments, including *Planctomycetota*, *Flavobacteriales*, *Oceanospirillales*, *Phycisphaerales*, *Zetaproteobacteria*, and *Candidatus* Marinibacteria (Supplementary Data S1). Considering that M1–M4 represent DLH enzymes that cannot degrade PET (as shown by the *in vitro* assays in Supplementary Fig. S9), our results support that although genes encoding pseudo-PETase enzymes are widespread across various bacterial taxa, only *Pseudomonadales* have primarily used these as a basis to develop PET-degrading M5 variants in marine environments.

## Discussion

In this study, we developed a motif-based classification system to distinguish functional PETases from non−/pre-functional variants or pseudo-PETases that utilize different substrates. The identified PETase-defining M5 motif incorporates structural elements essential for efficient substrate recognition and catalysis. The functionality of the M5 motif was experimentally confirmed, as only PETase variants containing this specific motif had measurable PET degradation activity, achieving 25%–50% of the efficacy observed for the *Is*PETase (*Is*-WT).

The M5 motif imposes strict structural and functional constraints, requiring not only the presence of the full catalytic triad but also specific amino acid residues essential for substrate binding and enzyme stability. These features make the M5 motif a far more selective criterion than the broadly defined DLH domain, which has been widely used in previous studies to search for potential PETases. This stringency results in a more restricted but functionally relevant set of sequences. For instance, applying the Hidden Markov Model (HMM)-based search method for putative PET hydrolases proposed by Danso and colleagues [29] to our dataset of 75 sequences containing DLH domain, we identified 37 candidate sequences, including all 23 that met our M5 motif criteria, as well as an additional 14 sequences with lower motifs. While this non-stringent search approach can expand the pool of potential PETase-encoding genes and increase the observed taxonomic diversity (see M4 variants in Supplementary Data S1), it also introduces uncertainty about functional capacity. The fact that only genes encoding enzymes with a complete M5 motif exhibited PET-degrading activity indicates that many proteins containing the DLH domain resembling PETase sequences at the structural level are likely adapted to act on different substrates or perform other biological functions. Indeed, the DLH domain is part of a broad family of α/β-hydrolases known to act on a range of substrates, including lactones, esters, and other xenobiotics [62]. Without the specific structural refinements encoded by the M5 motif—such as the conserved catalytic triad and substrate-stabilizing residues—these proteins do not possess the biochemical features necessary for effective PET degradation.

The application of this stringent criterion to ocean metagenomes resulted in a restricted taxonomic distribution, with M5-PETase variants primarily detected within the order *Pseudomonadales* and the phylum *Gemmatimonadota*. However, if we extend our analysis to a broader dataset of 80,000 DLH domain-containing sequences from UniProt—spanning ecosystems beyond the marine environment—we retrieved 379 compliant sequences (compared to 505 putative PETase sequences identified via HMM search) that span a wider range of bacterial taxa, such as *Actinomycetes* and *Betaproteobacteria* from terrestrial ecosystems, as well as *Gammaproteobacteria*/*Pseudomonadales* in both marine and non-marine ecosystems [63–65]. This demonstrates that our filtering approach does not inherently exclude PETases from diverse taxonomic groups but rather captures those sequences that most closely conform to the structural requirements for PET degradation. It also highlights a clear compositional shift in the microbial communities harboring functional M5-PETase variants, depending on the ecosystem, with marine environments predominantly enriched in *Pseudomonadales*, whereas terrestrial systems exhibit broader taxonomic representation [29, 63, 64]. The observed prevalence of M5-PETase genes and transcripts within *Pseudomonadales* can be explained by a combination of biological and environmental factors that contribute to their ecological success in marine ecosystems [66] and in degrading plastics [67]. A key factor is their high genetic adaptability in response to environmental pressures facilitated by a rapid mutation rate and horizontal gene transfer [68]. Another contributing factor is the pre-adaptation of *Pseudomonadales*, particularly *Pseudomonas* species, to organic pollutants. The structural similarity between PET-derived compounds, synthetic polymers and aromatic hydrocarbons may have facilitated the development of pseudo-PETase enzymes in different variants [69], likely via functional convergence.

In addition to the *in vitro* functional assay, microcosms inoculated with marine bacteria encoding M5- and non-M5-PETases were used to validate the predictive power of the motif-based classification. Beyond providing proof-of-concept for our classification framework, the *in vivo* assays revealed how the M5-PETase-encoding ISS-1242 strain degraded and altered PET films but did not use such substrate as the sole carbon source, as *I. sakaiensis* does [7]. A possible reason may be the absence of the MHETase-like gene, which enables them to use PET as an alternative carbon and energy source to grow [70]. A bioinformatics scan failed to detect potential MHETase-like genes in the genomes of the three isolates, even though they were detected in *Pseudomonas* from marine metagenomes (search in NCBI, Supplementary Table S4). However, the extended presence of MHETase-like genes in marine microbial communities from samples of the *Tara* Oceans and the Malaspina Expedition (Supplementary Fig. S17 and Data S5) suggests that plastic degradation in the oceans may rely on consortia of microorganisms with synergistic activities distributed over different bacterial classes in the same habitat. The composition of these communities is indeed dynamic and can vary based on several factors, such as the type of plastic, location, and surrounding environmental conditions [71–73]. Their associated microbial guild is consistently dominated by heterotroph members of *Pseudomonadota* (formerly *Proteobacteria*, such as *Gammaproteobacteria* and *Alphaproteobacteria*) [21], with a particular abundance of *Pseudomonadales* [20] that alone have been reported to degrade 35 different plastic types [64]. Beyond their functional role in plastic degradation, the microbial colonization dynamic and succession of PET and PET-derived substrates as the sole carbon source revealed that several taxonomic groups are pioneers, whereas *Pseudomonas* are only middle or late colonizers [69, 74], possibly because they lack the capacity to grow only by using PET in a relatively short time, as observed in our experiments. Thus, PETase-containing bacteria may utilize PET as an expanding resource in the deep ocean, where organic substrates are notably diluted [26], even if effective degradation and depletion of this “unusual” carbon source (and other plastics) depend on the collaborative presence of diverse bacteria [74, 75].

The widespread presence of PETase genes and their transcripts (mRNA) across oceans (Fig. 4 and Supplementary Fig. S19), combined with the acute carbon limitation in bathypelagic waters [26], suggests that exposure to plastic [76] may trigger the spontaneous evolution of this metabolic trait in ocean microbiomes. However, we acknowledge that other environmental factors, such as temperature and pH, may shape the distribution and expression of such enzymes, their intrinsic properties, and overall performance. Co-occurring stressors such as heavy metal contamination, hydrocarbon pollution, or eutrophication-related shifts in nutrient availability and oxygen levels can indeed significantly influence microbial community composition and functional gene dynamics. These factors may act synergistically or antagonistically with plastic pollution, potentially selecting microorganisms with broader metabolic versatility, including those capable of degrading PET.

While the naturally occurring PETases remain far from matching the catalytic efficiency of engineered high-performance variants [9, 14, 42], their functional validation (M5 motif confirmed *in vitro* and *in vivo*) and transcriptional activity (as detected by metatranscriptomic analyses) provide strong evidence that exposure to plastic-derived substrates is shaping microbial metabolic pathways across oceans. However, a major limitation is the lack of DNA-derived datasets from ocean samples collected prior to the widespread introduction of plastic pollutants. Although we searched publicly available metagenomic archives for such historical data, the oldest accessible datasets date back only to the early 1990s—by which time pseudo-PETase sequences, including M5-compliant variants, were already detectable. This prevents us from directly assessing whether the emergence or enrichment of PET-degrading M5-DLH domain enzymes was a consequence of exposure to plastic pollution. Future efforts to retrieve and sequence well-preserved, archived marine samples from the pre-plastic era could offer unprecedented insight into the temporal dynamics, emergence, and potential anthropogenic selection of functional M5-PETases. Such data will help determine whether motifs like M5 evolved as a direct response to plastic introduction or if they represent pre-existing enzymatic adaptations co-opted under new environmental pressures.

## Supplementary Material

Alam_et_al_Supplementary_Information_wraf121

Alam_et_al_Additional_file_Data_S1_wraf121

Alam_et_al_Additional_file_Data_S2_wraf121

Alam_et_al_Additional_file_Data_S3_wraf121

Alam_et_al_Additional_file_Data_S4_wraf121

Alam_et_al_Additional_file_Data_S5_wraf121

Alam_et_al_Additional_file_Data_S6_wraf121

Alam_et_al_Additional_file_Data_S7_wraf121

Alam_et_al_Additional_file_Data_S8_wraf121

Alam_et_al_Additional_file_Data_S9_wraf121

## Data Availability

Data analysed here are either available from the original studies or in the following repository: *Tara* Ocean shotgun sequence data are available from ENA (PRJEB1787); MD sequence data are available from ENA (PRJEB40454); MP sequence data are available from ENA (PRJEB52452). For the expression of genes at the metatranscriptome level, we used the *metaT* gene profile from https://www.ocean-microbiome.org/. Genomes of the three *Pseudomonas* isolates are available from NCBI BioProject PRJNA1039609.

## Code availability

Codes associated with this manuscript and detailed instructions for utilizing them will be provided upon request.
