# Supplementary material for: Widespread distribution of bacteria containing PETases with a functional motif across global oceans
Source: ISME J. 2025 Jun 10;19(1):wraf121. doi: 10.1093/ismejo/wraf121 (PMC12224614; doi:10.1093/ismejo/wraf121)
Supplement: Alam_et_al_Supplementary_Information_wraf121 [file alam_et_al_supplementary_information_wraf121.pdf]

Supplementary Information of

Widespread distribution of bacteria containing PETases with a functional motif across global oceans

Intikhab Alam[1#*], Ramona Marasco[2#], Afaque A. Momin[3#], Nojood Aalismail[4], Elisa Laiolo[4], Cecilia Martin[4], Isabel Sanz-Sáez[5], Begoña Baltá Foix[5], Elisabet L. Sá[5], Allan Kamau[1], Francisco J. Guzmán-Vega[3], Tahira Jamil[1], Silvia G. Acinas[5], Josep M. Gasol[5], Takashi Gojobori[2,6], Susana Agusti[4], Daniele Daffonchio[2,7,$], Stefan T. Arold[3,$], and Carlos M. Duarte[4*]

[1]Center of Excellence for Smart Health, Computer, Electrical and Mathematical Science and Engineering Division, King Abdullah University of Science and Technology, Thuwal, Saudi Arabia

[2]Biological and Environmental Science and Engineering Division, King Abdullah University of Science and Technology, Thuwal, Saudi Arabia

[3]Center of Excellence for Smart Health, Biological and Environmental Science and Engineering Division, King Abdullah University of Science and Technology, Thuwal, Saudi Arabia

[4]Marine Science Program, Biological and Environmental Science and Engineering Division, King Abdullah University of Science and Technology, Thuwal, Saudi Arabia

[5]Departament de Biologia Marina i Oceanografia, Institut de Ciències del Mar-CSIC, Pg. Marítim de la Barceloneta 37-49, 08003, Barcelona, Spain

[6]Department of Life Sciences, National Cheng Kung University No.1, University Road, Tainan City, Taiwan (R.O.C.)

[7]Department of Agriculture Forestry and Food Sciences, University of Turin, Grugliasco, Turin, Italy

*Corresponding authors. Intikhab Alam, Email: intikhab.alam@kaust.edu.sa; Carlos Duarte, Email: carlos.duarte@kaust.edu.sa

[#]Intikhab Alam, Ramona Marasco, and Afaque A. Momin shared first authorship on this work.

[$]Daniele Daffonchio and Stefan T. Arold contributed equally to this work.

Supplementary Methods

Methods S1. Identification of marine bacterial strains encoding for PETase gene. Using available PETase sequences retrieved from marine metagenomes (PETase from this study; **Data S1**) and *Pseudomonas aestusnigri* [1] as a positive control, primers targeting a portion of the PETase gene were designed. The sequences for the PETase primers are the following 214F (5'-GTCAGCGGCTTTGGCGGCGG-3') and 729R (5'-ACAAAGGCCTTGTCGATG-3'). From the marine heterotrophic culture collection (MARINHET) [2], six *Halopseudomonas* (formerly *Pseudomonas* [3, 4]) strains were tested (**Table S3**). DNA was extracted starting from 35 mL of liquid culture grown into Zobell medium (5 g peptone, 1 g yeast extract and 15 g agar in 750 mL of 30 kDa filtered seawater and 250 mL of Milli-Q water) using DNeasy PowerSoil Kit. The concentration of extracted DNA was determined by spectrophotometry using NanoDrop (Thermo Fischer). The PCR was conducted with the newly designed PETase primers following the thermal protocol used to amplify the 16S rRNA gene [2]. The length of the products was verified by electrophoresis in agarose gel (with 100 ml of TAE solution 1× (buffer), 1.2 g of agarose and 5 µL of SYBR™ Safe DNA Gel Stain) and all were of the estimated size of about 515 bp. Purification and OneShot Sanger sequencing of PETase gene products was performed by Genoscreen (Lille, France). ChromasPro 2.1.8 software (Technelysium) was used for manual cleaning and quality control of the sequences. The results were edited with the BIOEDIT program and then aligned to available sequences with the GenBank database, National Centre for Biotechnology Information (http://www.ncbi.nlm.nih.gov/) by BLASTn and BLASTp.

Method S2. Bacterial genome sequencing. Bacterial cultures were grown at 24 °C for 24 h in MB 2216 medium. 1 mL of a 10^9 cells/mL solution was prepared and used to extract genomic DNA with the Maxwell RSC Automated Nucleic Acid Purification system and the Maxwell® RSC Cultured Cells DNA kit (Promega). DNA concentration was quantified using the Qubit® dsDNA BR Assay Kit (Thermo-Fisher Scientific), and the quality was assessed by electrophoresis on 0.8% agarose gel and Bioanalyzer 2100 (Agilent). Genomic DNA of the ISS-721, ISS-1225 and ISS-1242 strains was sequenced at KAUST Bioscience Core Lab using PacBio RS2 sequencer (Pacific Biosciences). The PacBio reads were assembled using the microbial assembly workflow available in PacBio's SMRT analysis tools and submitted to NCBI under project accession PRJNA1039609. Genome annotation was done using the KAUST Metagenomic Analysis Platform (KMAP). Complete genomes were compared against the closest species *Halopseudomonas pachastrellae* for which genomes were available (NCBI assembly accession GCF_900114765 and GCF_001989375) [4, 5], using Average Nucleotide Identity implementation in dRep software [6].

Supplementary Results

Result S1. Structural analysis and efficiency scoring of PETases. High-confidence homology models were produced for all putative PETases by SWISS-MODEL[7], based on up to 50% sequence identity with the crystal structure of the *Is*PETase in a complex with HEMT (PDB ID 5xh3). The structural analysis of potential PETases is described in detail in the following paragraph, based on the example of the PETase_02. This selected putative PETase contains the catalytic triad (S160, D206, H237) and the disulfide bond linking residues C203 and C239, demonstrated to be essential for PETase function[8, 9] (**Fig. S6**). In addition, the higher-scoring PETases harbour two modifications that were shown to increase the catalytic activity of *Is*PETase[10], namely R90A and L117F. R90A is thought to reduce the steric hindrance around the active site and increase the hydrophobic area to facilitate the accumulation of substrate near the substrate-binding cleft. L117F stabilises Y87 (F87 in this case), which enhances the interaction between the enzyme and the substrate, as shown by a lower Michaelis constant, *Km,* of the L117F mutant as compared to the wild-type PETase. The S214H variant found in these PETases has been seen to reduce PETase activity, possibly because the larger His restricts the movement of the adjacent W185[8]. Also, other variants that have not been experimentally tested were found in these PETases (**Data S2**), and their effect on the catalytic activity of the protein remains uncertain. The variants Y87F, T88V and S238Y/F increase the hydrophobicity around the active site, which is expected to be beneficial for PETase activity, as were other substitutions increasing the hydrophobicity of this region, as they may facilitate the interaction of the enzyme with the substrate. The R280Q substitution in most of the higher-scoring variants is conservative and not expected to influence catalysis, and other important positions such as W159, W185 and M161 remain unchanged in these variants. Overall, the compound effects of these modifications make these variants good PETase candidates with a chance of having a PET degrading activity close to or potentially better than *Is*PETase. However, the small difference between the scores of many of the analysed sequences reflects the lack of information about the numerous existing variants in these sequences and how they may influence PETase activity. It is also important to note that these scores cannot be taken as an actual measure of PET-degrading efficiency but are merely the result of a comparison of which sequences seem to be most similar to *Is*PETase and which of them could potentially be better or worse adapted based on the presence of favourable or inhibiting mutations.

Result S2. Assembly and annotation of genomes. High-quality assemblies were achieved for all three *Pseudomonas* strains, namely ISS721 (Internal ID PSAMP1), ISS1225 (Internal ID PSAMP2), and ISS1242 (Internal ID PSAMP3). ISS721 and ISS1225 yielded single-contig chromosome assemblies of approximately 4.39 Mb in size, while ISS1242 assembled into a near-complete genome, with a main contig (~4.13 Mb) and two smaller contigs. These genomes are submitted to NCBI under bio-project PRJNA1039609.

Each genome harboured a PETase-like gene, although the quality and integrity of the PETase motifs varied (**Fig. S10**):

(i) ISS721 contains a fragmented PETase pseudogene split across two loci. When conceptually joined, the sequence suggests the presence of an M4-like motif, though its disrupted nature likely compromises functionality.

(ii) ISS1225 exhibits a partial PETase-like sequence containing only the M3 motif, with several critical regions either missing or degraded, suggesting limited enzymatic activity.

(iii) ISS-1242 encodes a full-length PETase-like enzyme with a complete M5 functional motif, indicating a strong potential for PET degradation. PHI-BLAST analysis confirmed the presence of the canonical M5 motif with high confidence.

We further assessed the presence of the secretion signal peptide in the isolates using the SignalP 6.0 web server (**Fig. S11)**. The results predict the presence of a secretion signal peptide in ISS-1242 with a likelihood of 0.9976 and a probability of 0.9699 with a cleavage site between residue 25 and 26; no secretion signal peptide has been detected in ISS-1225 and ISS-721 strains.

Supplementary Figures

Figure S1. The locations of the sites from which the metagenomes used in this work originated are indicated in the map. Sites from the *Tara* Oceans and Malaspina expeditions are represented in blue and red, respectively.

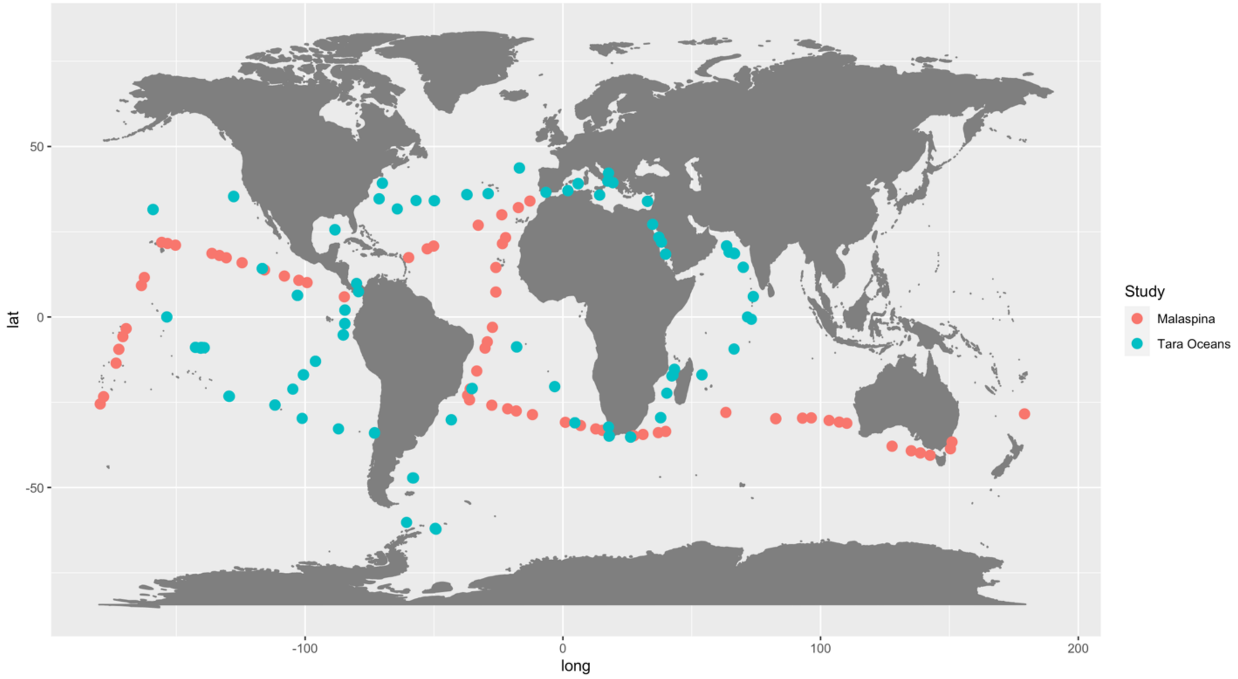

Figure S2. (**A**) SDS page gel for recombinantly produced eleven PETases, with motifs ranging from M1 to M5, including the M5 PETase gene of ISS-1242 (PETase12.M5) showing >90% purity. (**B**) Differential scanning fluorimetry measurements for *Is*-PETases and PETases with M5 and M4 motifs showed melting temperatures (T_m) between 47-51 °C.

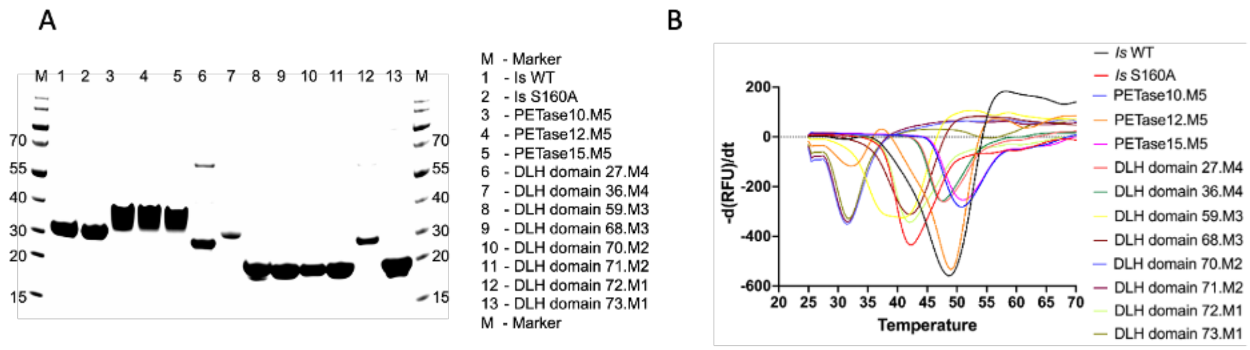

Figure S3. Comparison of genomes from the three isolates (ISS-1242, ISS-1225 and ISS-721) vs closer related species *Halopseudomonas pachastrellae* (GCF_900114765 and GCF_001989375) using Average Nucleotide Identities. The similarity is over 96%, confirming that the isolates belong to *H. pachastrellae*, even if they form two separate clusters, one with ISS-1242 and the other with ISS-1225 and ISS-721, suggesting they represent different species in the same genus.

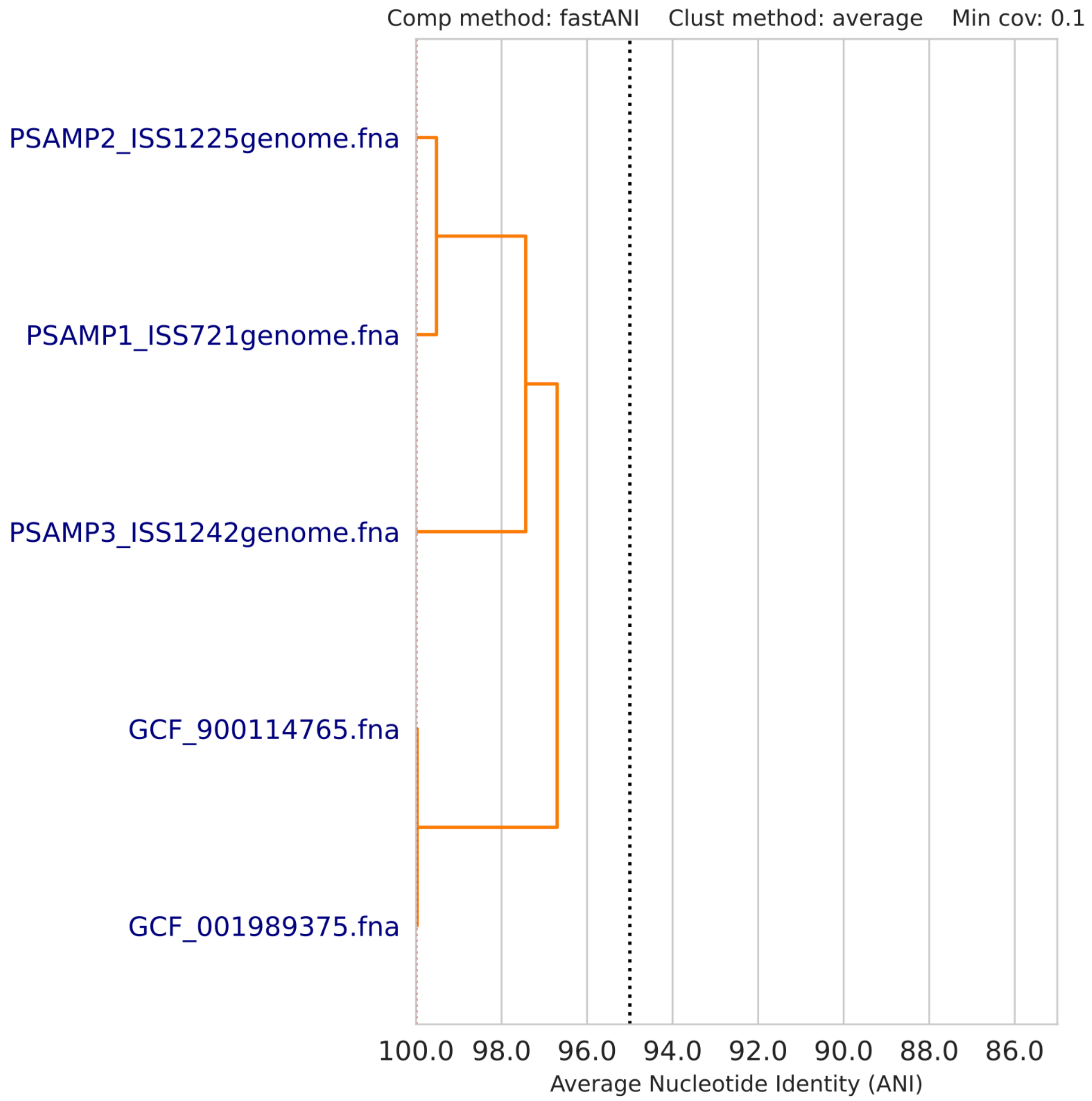

Figure S4. The degradation of the PET films was reported as a percentage of weight loss over time (days) for (**A**) ISS-721, (**B**) ISS-1225 and (**C**) ISS-1242 strains. Non-inoculated controls (NC) were also prepared for each of the media. The media used for the incubation are: (i) modified MB without C (mMB, the medium contained all components except yeast extract and peptone), (ii) modified MB with the addition of 0.01% of yeast extract (mMB+C), (iii) Red Sea water (SW) and (iv) and SW with the addition of 0.05% of yeast extract and 0.25% of peptone (SW+C). PET degradation in the non-inoculated controls is reported as the average of the values measured in the four media. The PET film degradation showed varying degrees of weight loss after 37 days of incubation. The NC did not show PET degradation (weight reduction, 0.43% ± 0.23%), as also observed for the three bacterial strains when incubated in the absence of extra C-sources (*i.e.*, mMB and SW; ISS-721= 0.59% ± 0.35%, ISS-1225 = 0.41% ± 0.49%, ISS-1242 = 0.97% ± 0.08%). The ISS-1242 strain showed the highest PET degradation values in the SW+C medium (3.7%), followed by the mMB+C medium (up to 1.9%). Strains ISS-721 and ISS-1225 showed a slight reduction of PET weight only in the SW+C medium.

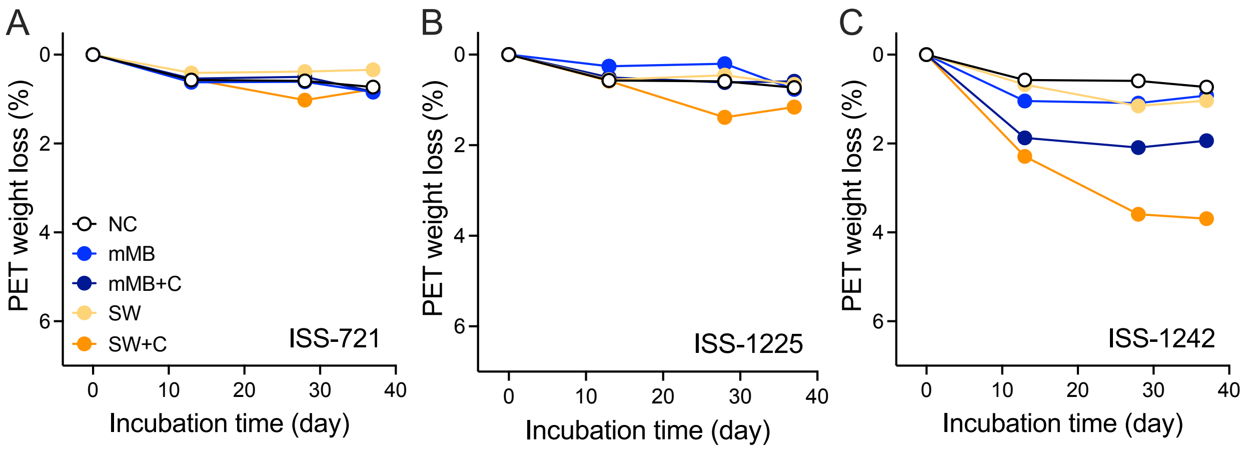

Figure S5. Molecular weight standards for BHET and TPA were used for the BHET degradation assay.

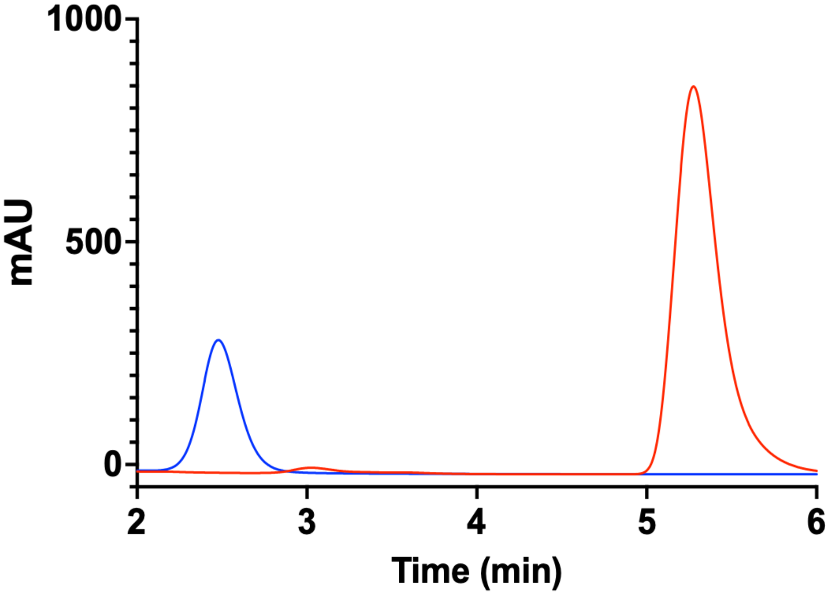

Figure S6. Illustration of protein structures representative of each motif category, with the corresponding motif M1 to M5 coloured in lime green. The models were obtained by SWISS-MODEL based on the crystal structure of *Is*PETase in complex with HEMT (PDB ID 5xh3) as a template. Key residues are shown as stick models, colour-coded as follows: red, the catalytic triad; blue, the Met residue involved in the oxyanion hole; dark grey: residues forming the aromatic clamp, where [YF]87 is part of both the aromatic clamp and the oxyanion hole.

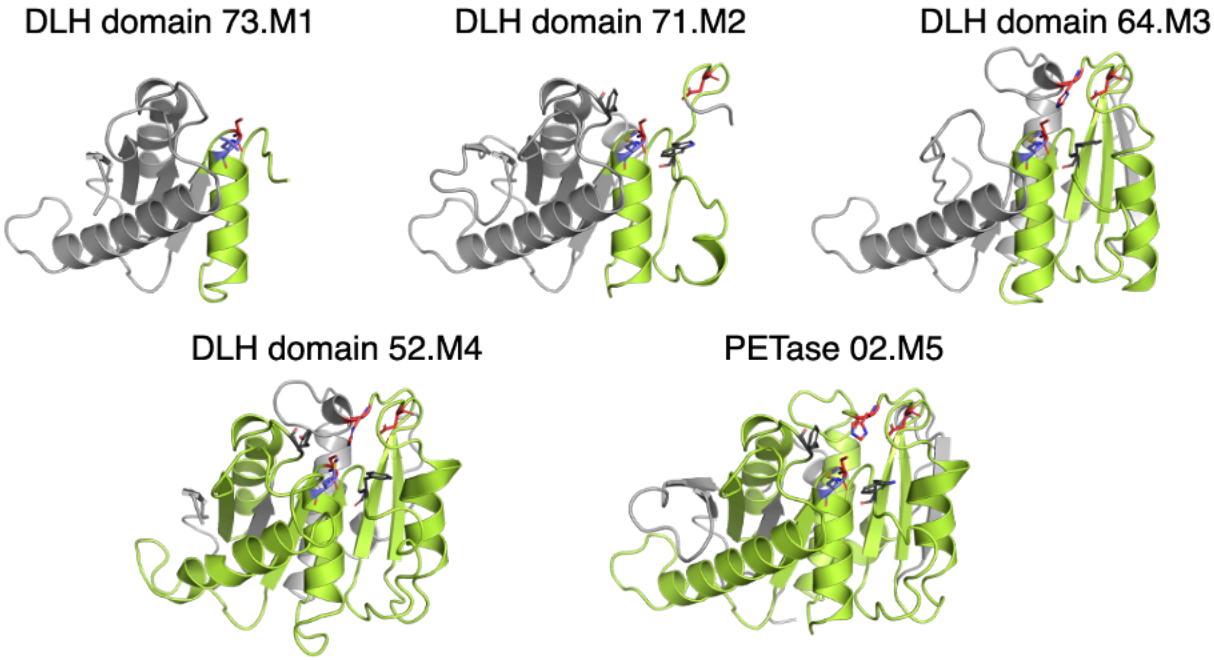

Figure S7. Predicted 3D models for M3, M4 and M5 PETases. Most of them conserve the full alpha/beta hydrolase motif from *Is*PETase. The motif regions are coloured orange, and the important residues captured by each motif are shown in red sticks.

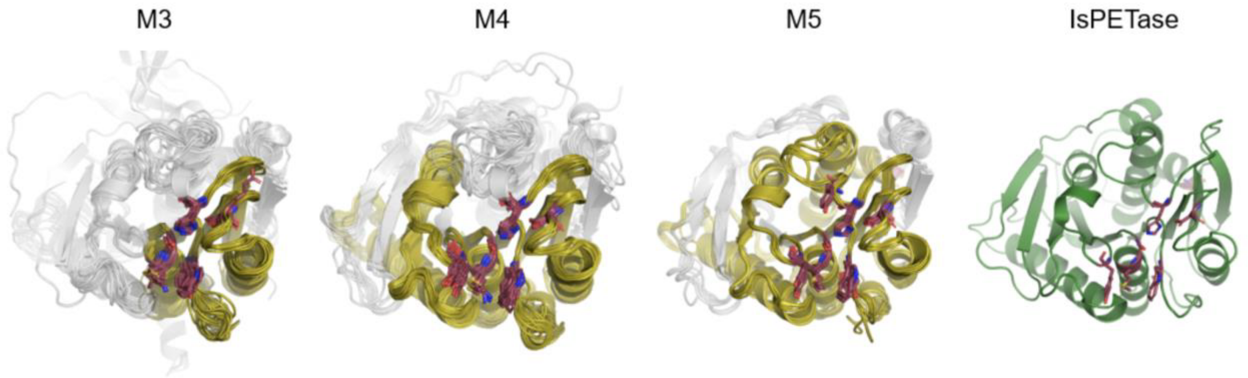

Figure S8. Alignments of ocean PETase variants assigned to motifs M3, M4, and M5 show conservation and variation in key residues, particularly catalytic tirade, aromatic clamp and oxyanion hole.

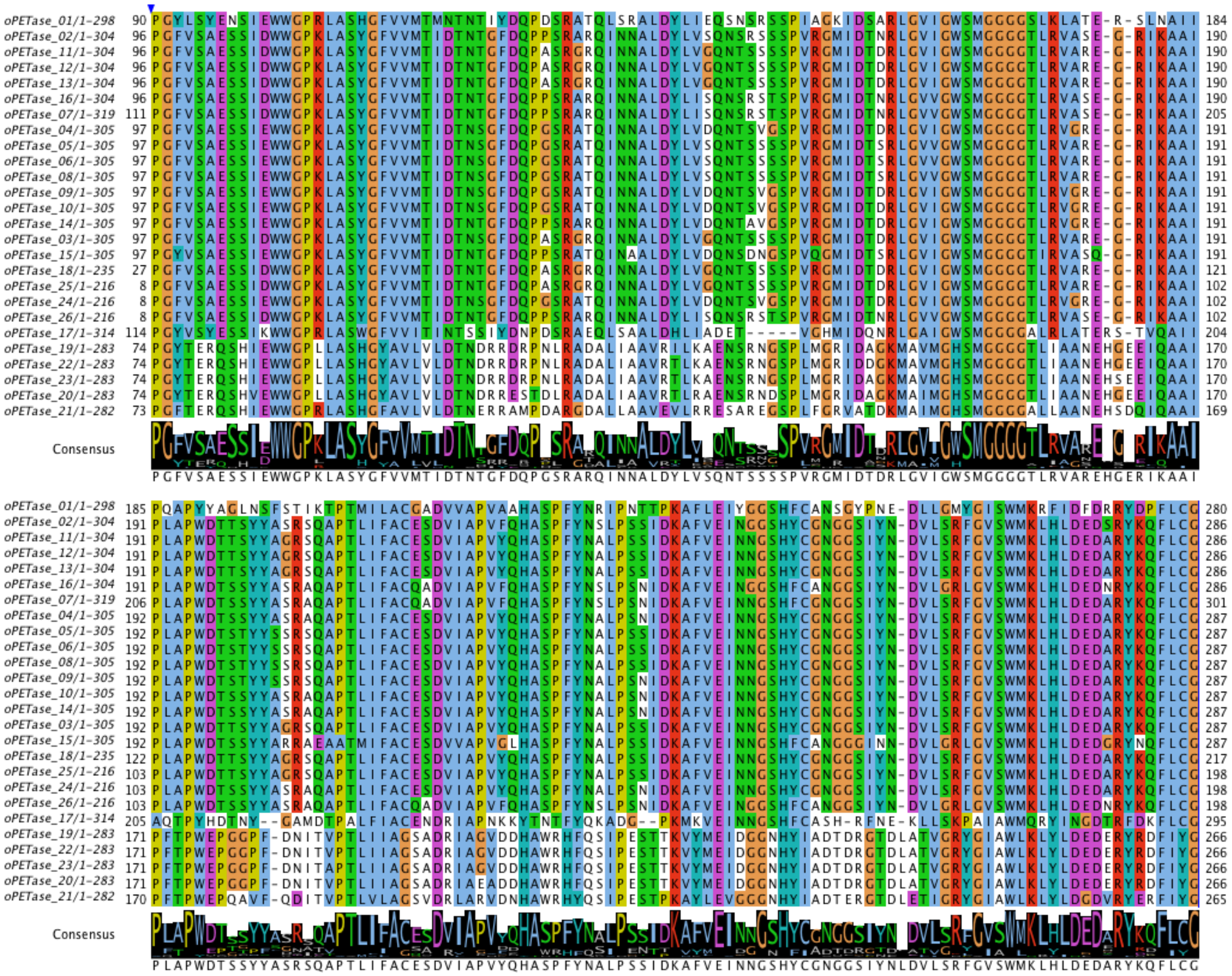

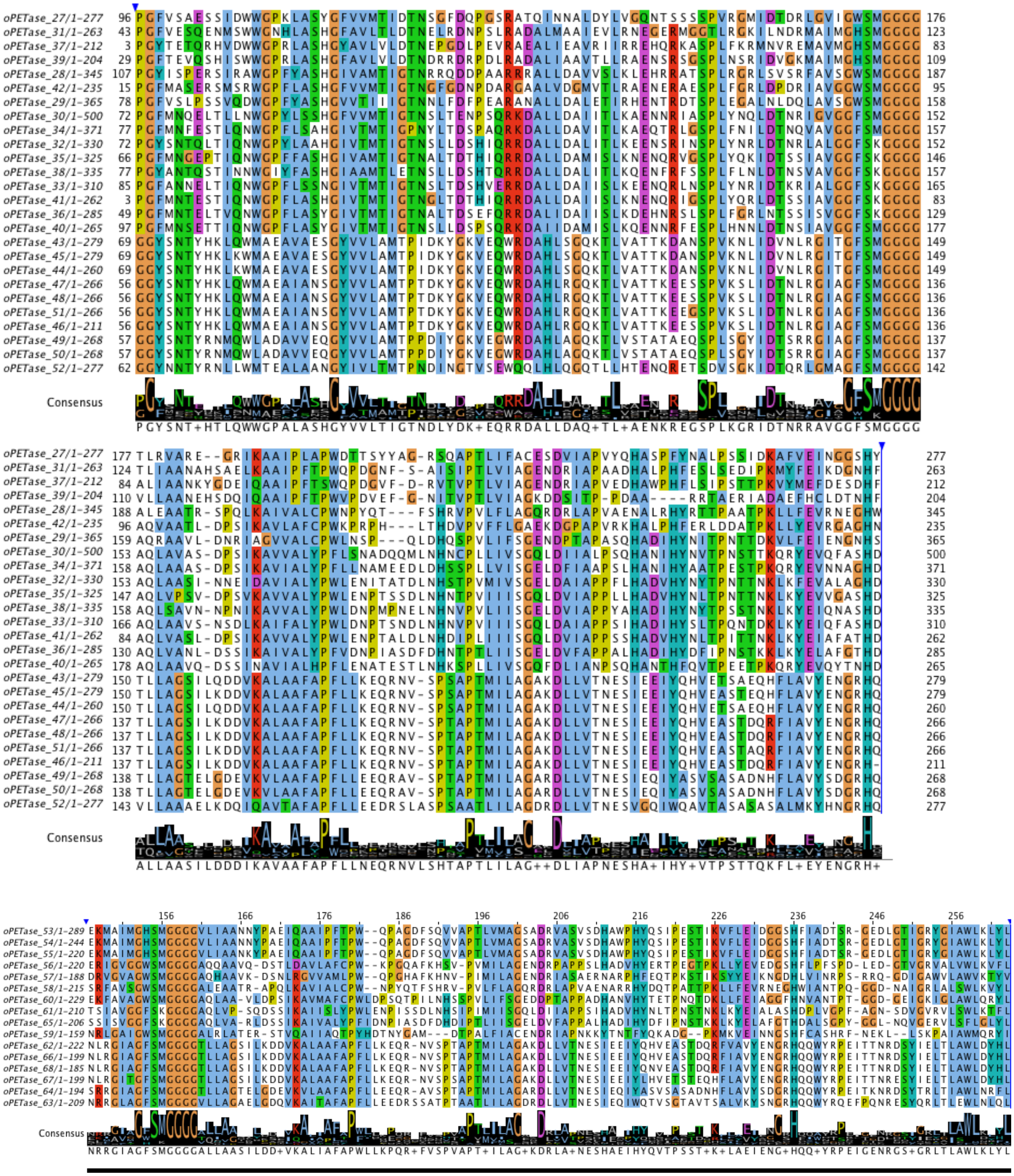
oPETase_27/1-277
oPETase_31/1-263
oPETase_37/1-212
oPETase_39/1-204
oPETase_28/1-345
oPETase_42/1-235
oPETase_29/1-365
oPETase_30/1-500
oPETase_34/1-371
oPETase_32/1-330
oPETase_35/1-325
oPETase_38/1-335
oPETase_33/1-310
oPETase_41/1-262
oPETase_36/1-285
oPETase_40/1-265
oPETase_43/1-279
oPETase_45/1-279
oPETase_44/1-260
oPETase_47/1-266
oPETase_48/1-266
oPETase_51/1-266
oPETase_46/1-211
oPETase_49/1-268
oPETase_50/1-268
oPETase_52/1-277
Consensus
oPETase_53/1-289
oPETase_54/1-244
oPETase_55/1-220
oPETase_56/1-220
oPETase_57/1-188
oPETase_58/1-215
oPETase_60/1-229
oPETase_61/1-210
oPETase_65/1-206
oPETase_59/1-159
oPETase_62/1-222
oPETase_66/1-199
oPETase_68/1-185
oPETase_67/1-199
oPETase_64/1-194
oPETase_63/1-209

Figure S9. Enzymes tested on PET film (**A**) or PET granules (**B**) over 14 days, followed by HPLC plots for each time step, showing *Is*-S160A and the M1-M4 DLH domain enzymes did not display significant activity in these assays.

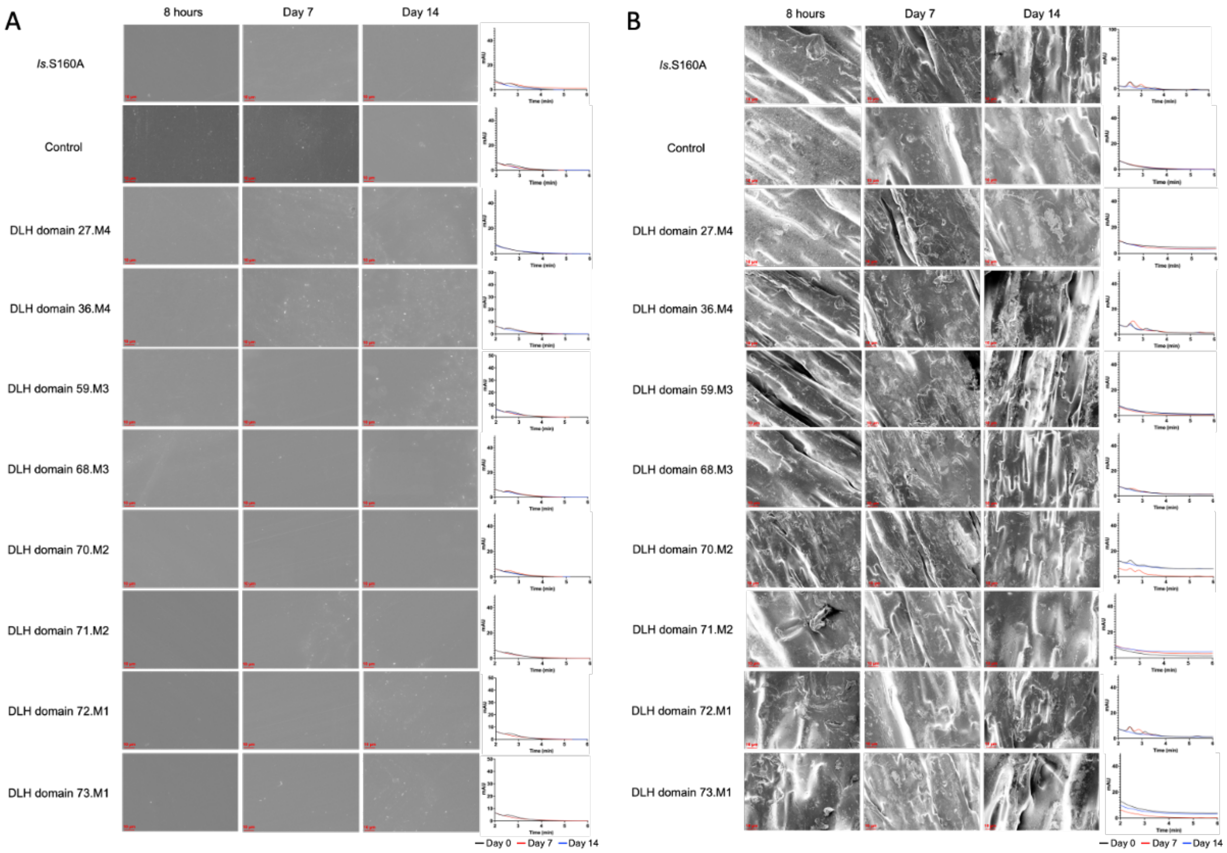

Figure S10. (**A**) AlphaFold2 3D models built for ISS-1242, ISS-721, and ISS-1225 show that ISS-721 and ISS-1225 contain partial PETase fold (lacking important C-terminal structural elements). (**B**) Sequence alignment for ISS-1242, ISS-1225 and ISS-721 shows that the ISS-1552 and ISSM721 lack important regions involved in PETase activity. The alignment is generated by Espript 3.0, and the secondary structure is shown for ISS-1242 on the top.

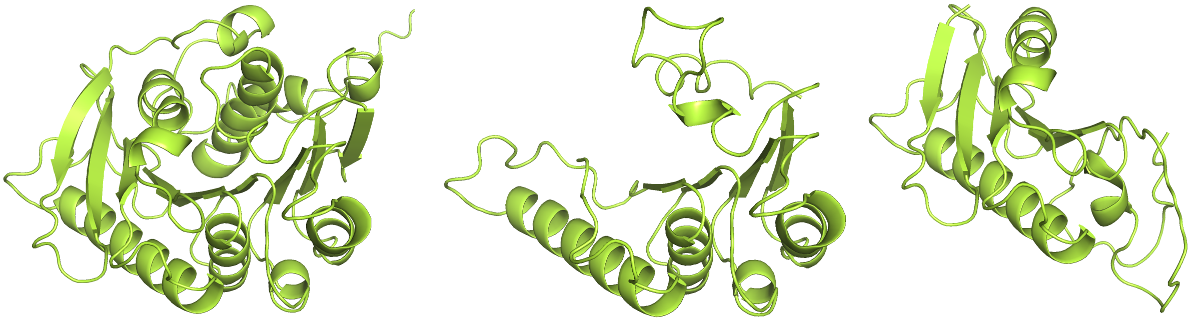

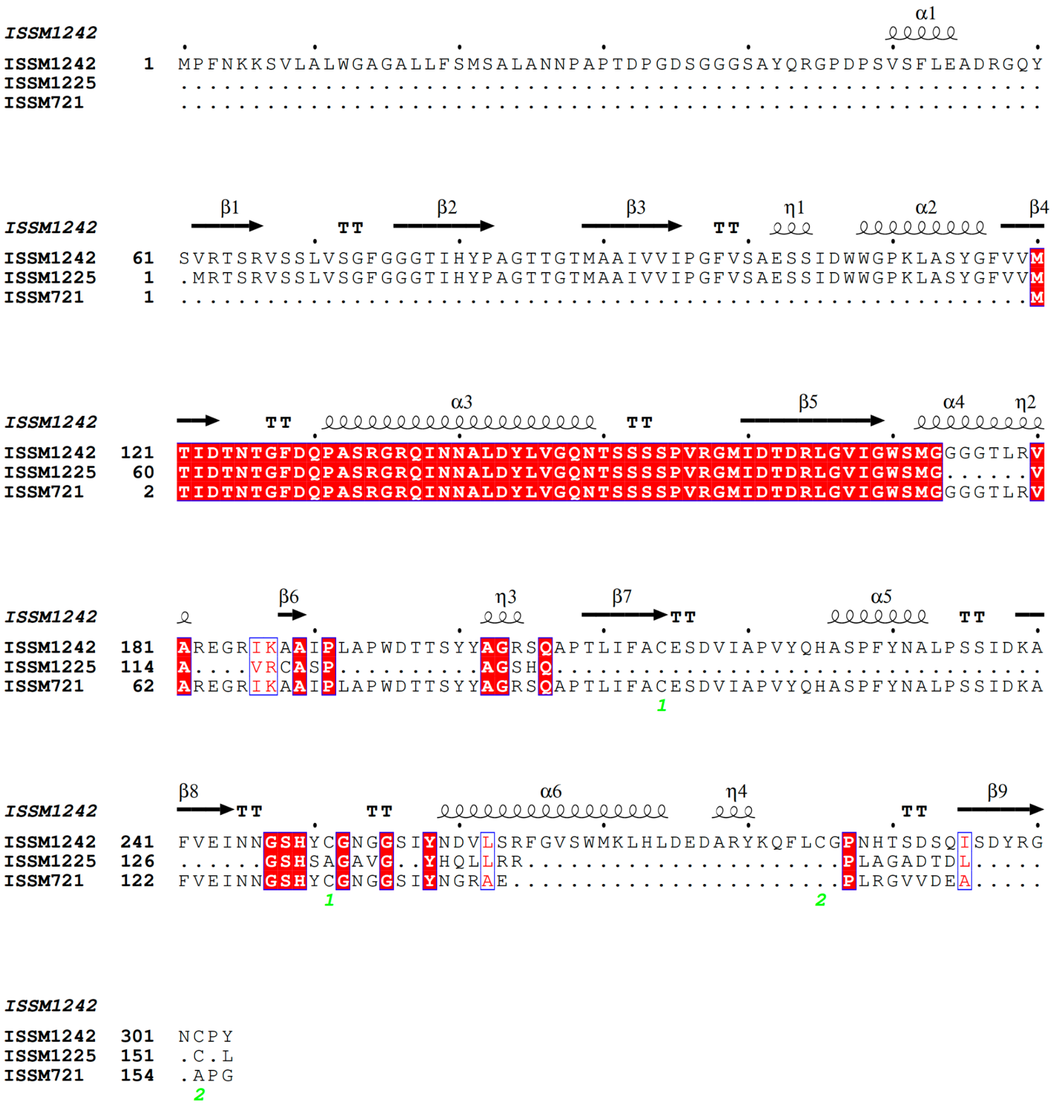

Figure S11. Signal peptide prediction plot generated using the SignalP 6.0 web server based on protein language models (LMs). The presence of secretion signal peptide is detected in (**A**) ISS-1242 strain but not in (**B**) ISS-1225 and (**C**) ISS-721 strains. The signal peptide was reported for Sec substrates cleaved by SPase I (Sec/SPI) in the n (n-terminal region - red), h (central hydrophobic region - orange), and c (c-terminal region - yellow) of the signal peptide. The cleavage site (CS - green) was found at position 25. Other (red dotted line) indicates sequence from Archaea, Gram-positive and Gram-negative bacteria.

A

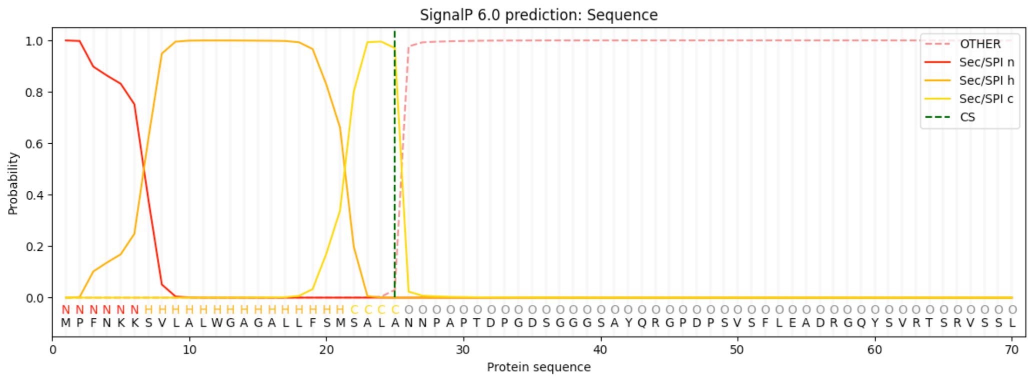

B

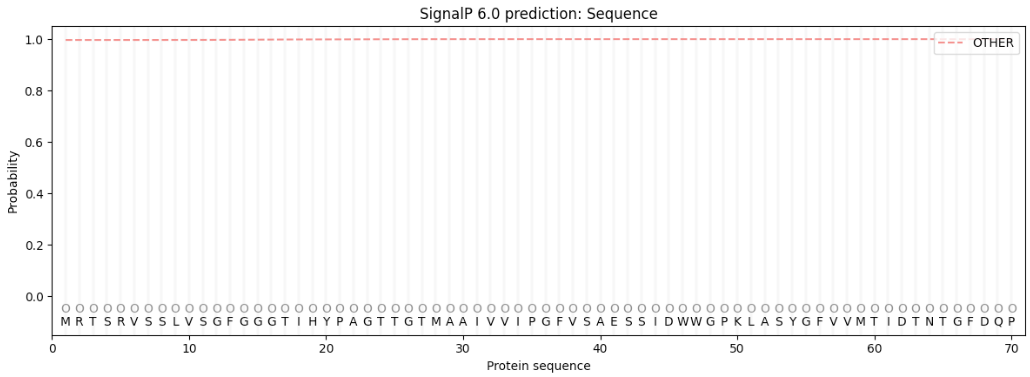

C

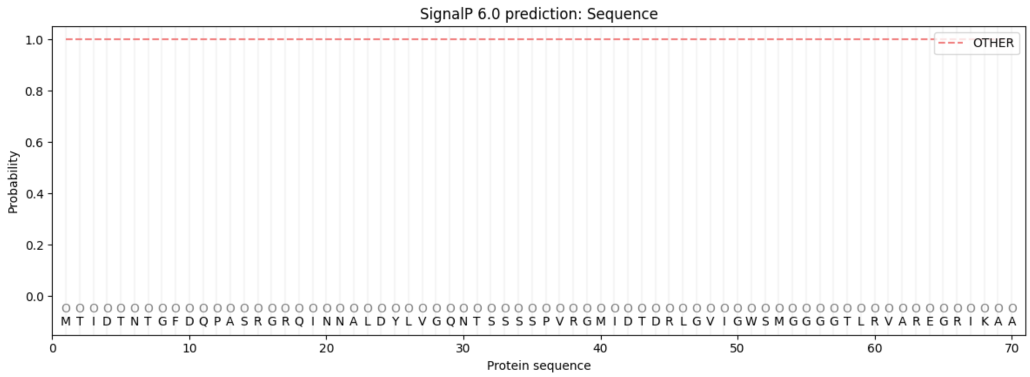

Figure S12. (**A** and **B**) Time course of PET film weight loss (%) by *Halopseudomonas* ISS-721 and ISS-1225 strains, respectively, along with the non-inoculated control (NC) in SW+C medium and *Escherichia coli* inoculated in M9+C medium. Data are reported as an average of three replicates, and their range of two distinct experiments conducted independently indicated as (I) and (II). *E. coli* microcosms were also included in the experiment (II). (**C**) HPLC profiles from the ISS-721 and ISS-1225 bacterial cultures that have been incubated with PET for 60 days from the experiment (I). Control refers to the medium and PET film non-inoculated with bacteria. Molecular weight standards are shown in **Fig. S6**.

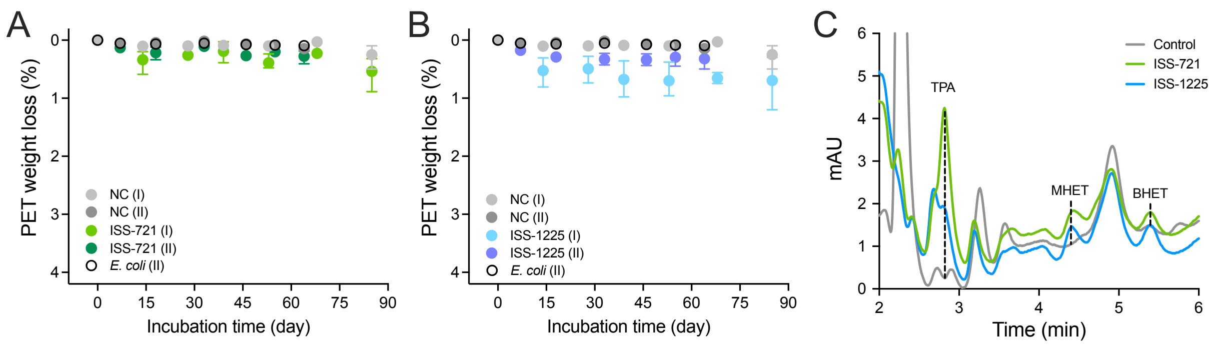

Figure S13. SEM images represent the surface of PET film incubated in the medium without bacteria after 60 days. Scale bars are reported in each panel.

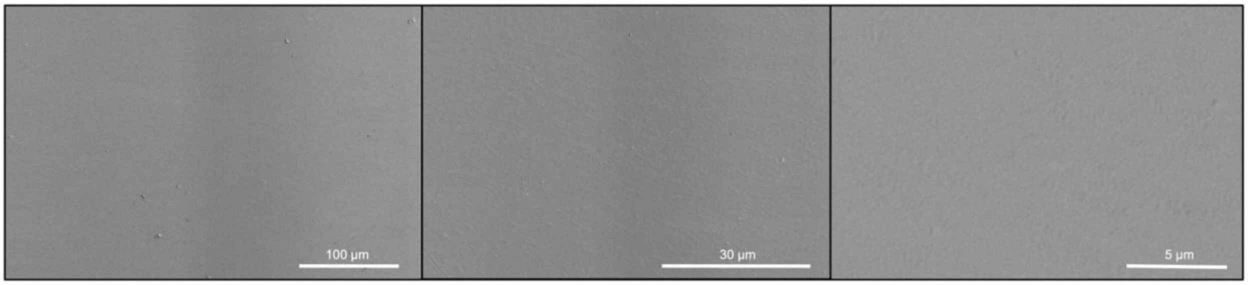

Figure S14. Global distribution of M5-PETase variants in marine metagenomes of (**A**) surface (0-100 m), (**B**) mesopelagic (200-1,000 m) and (**C**) deep waters (> 1000m). The abundance of PETase is normalised per abundance of the *recA* gene and expressed as FPKM.

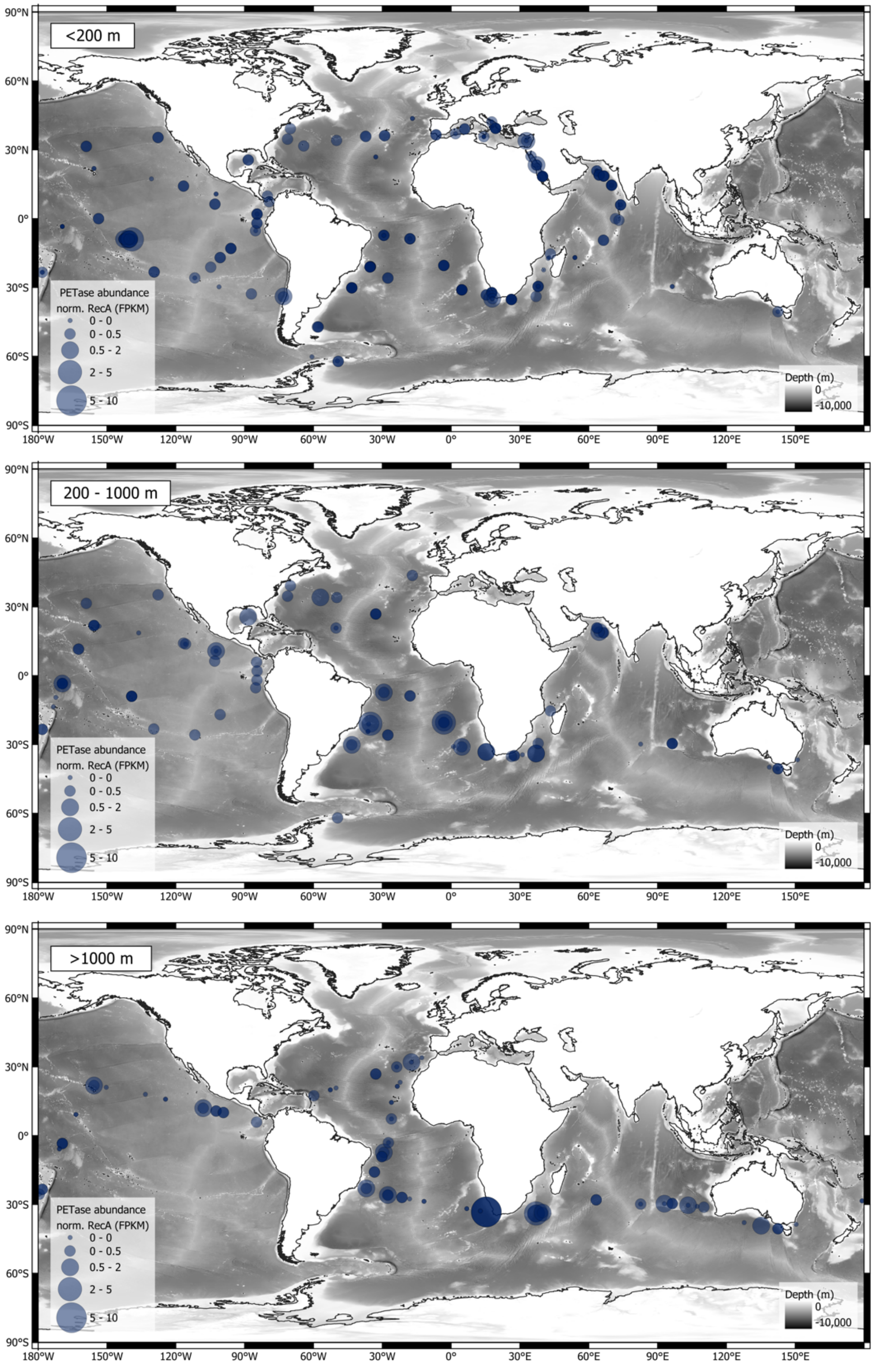

Figure S15. Relative abundance of M5-PETases across metagenomes. Data are reported as average (st.dev.) of M5-PETase abundance normalised per *recA* gene abundance and expressed as FPKM. Values are reported for depth categories.

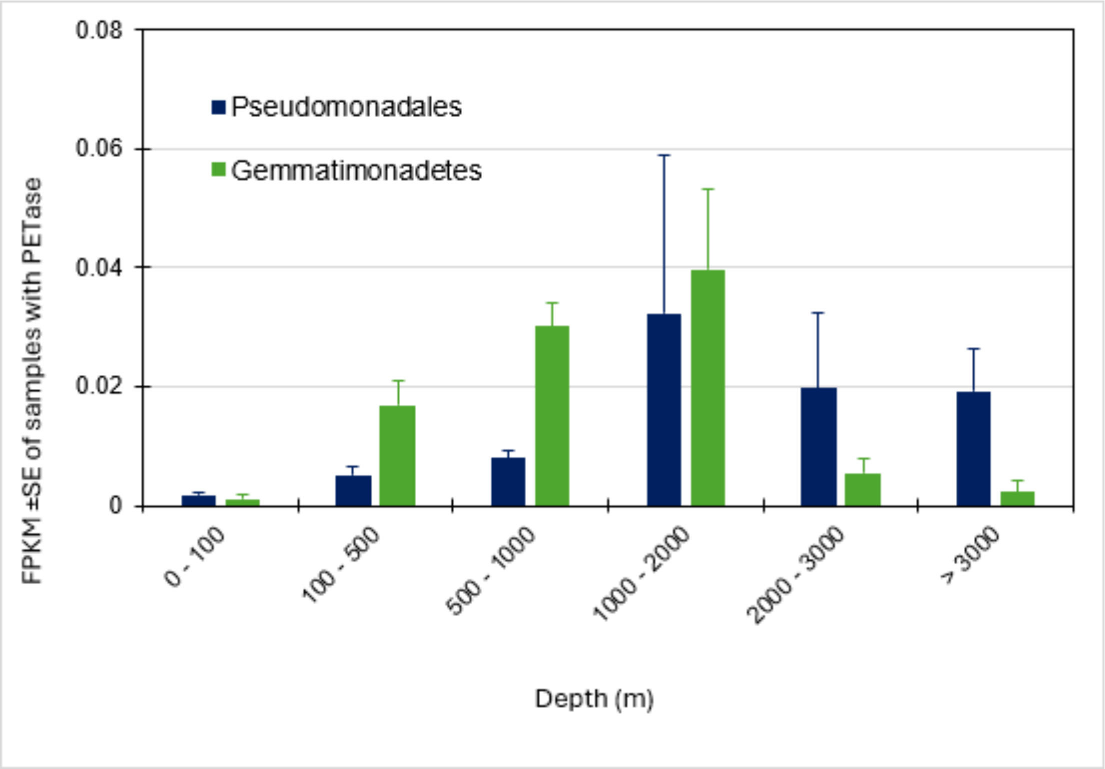

Figure S16. (**A-C**) Mean M5-PETase abundance (± SE), normalised per *recA* abundance expressed in FPKM across different filters (size range is expressed in µm) during the three *Tara* Oceans and Malaspina (MP and MD) expeditions. (**D-F**) Prevalence of samples where PETase occurred in each filter for the three expeditions.

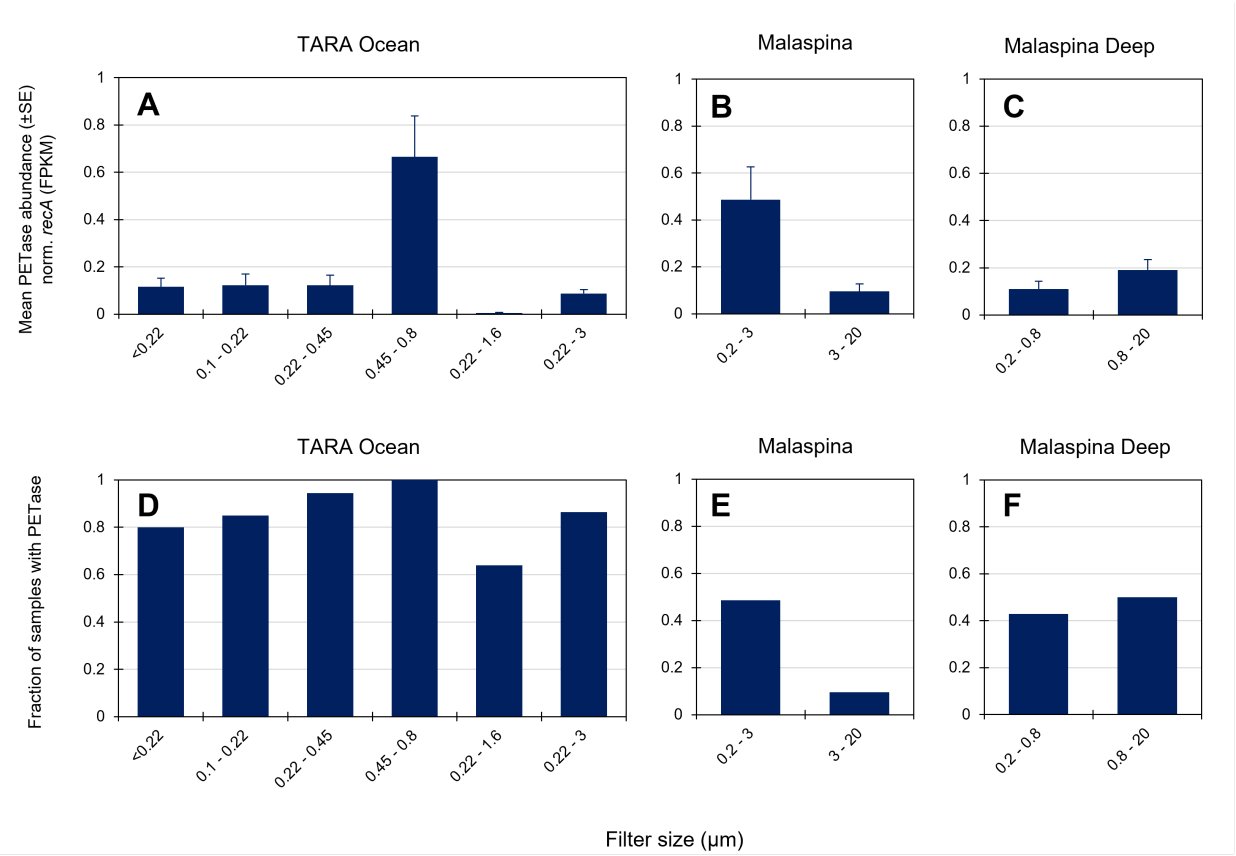

Figure S17. BLAST search for MHETase in the *Tara* Oceans and Malaspina data (https://tara-oceans.mio.osupytheas.fr). The distribution of MHETase across samples is reported in **Data S5**.

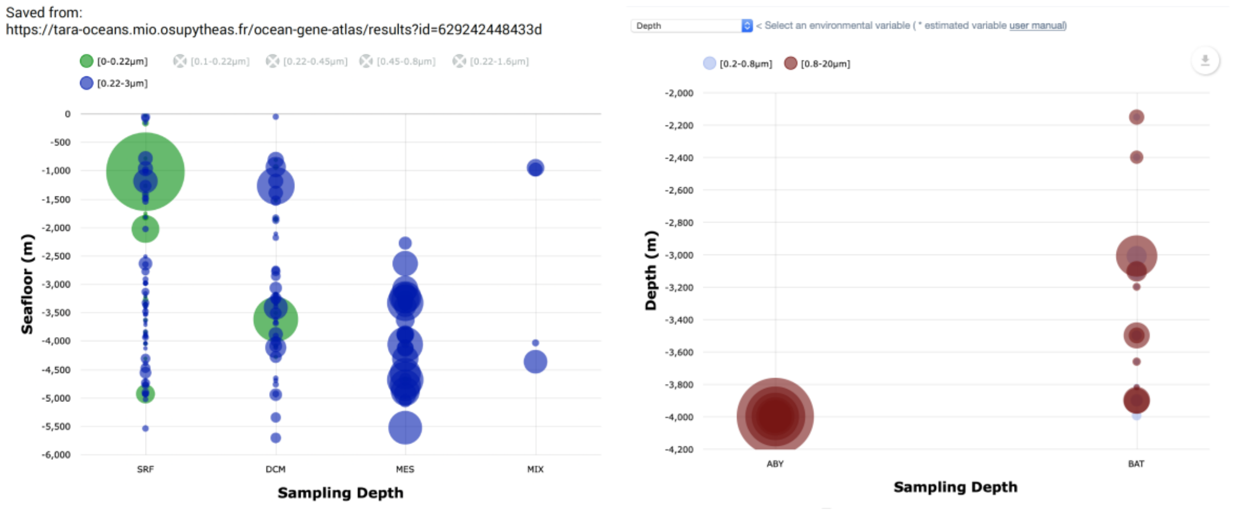

Figure S18. Map of the 816 records of association between plastic samples and M5-PETase samples based on thresholds applied (see **Methods** and **Data S6A**). Plastic samples are represented in red shades per legend, and abundance is expressed as g per km^2. The abundance of M5-PETase variants is represented in green shades per legend; values are expressed as M5-PETase abundance normalised per *recA* abundance in FPKM. Only M5-PETases associated with plastic sites are represented. These include the samples that fulfil the threshold of i) 5° radius distance from the plastic samples and ii) a depth of < 10 m (the maximum detected in this set of samples is 5 m).

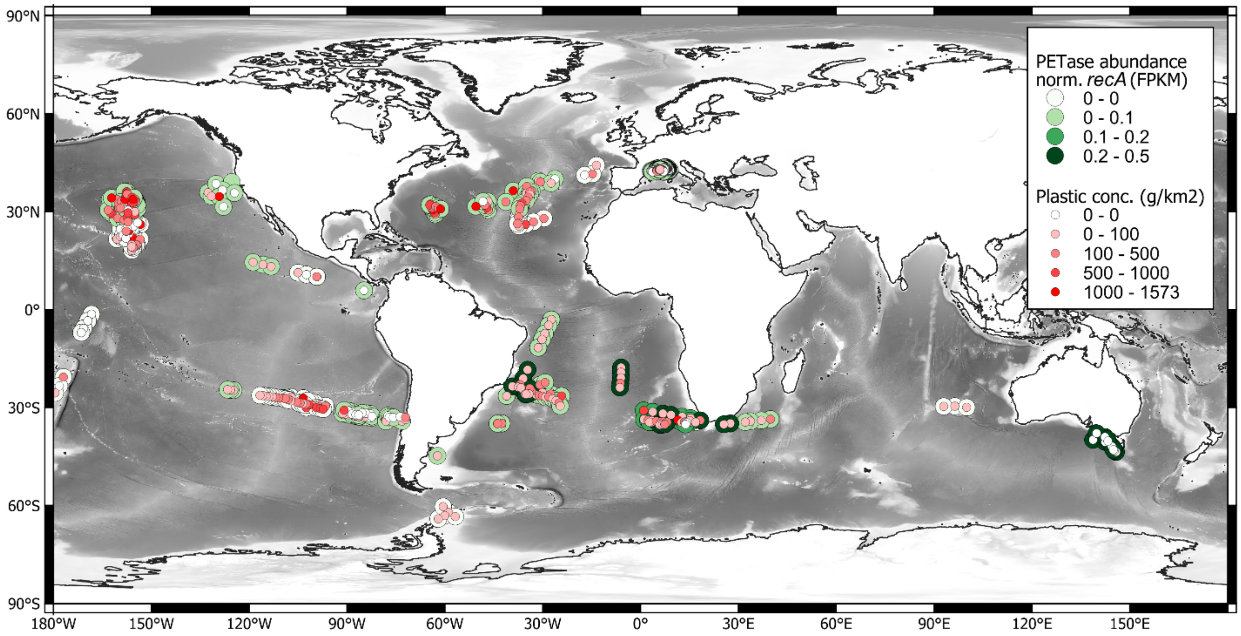

Figure S19. Distribution and prevalence of M5-PETase transcripts from the metaT dataset. (**A**) Locations of samples (*n* = 185) from which M5-PETase gene expression was retrieved across oceans and relative abundance of their transcript values normalised per abundance of *recA* gene transcripts (expressed as Fragments Per Kilobase of transcript per Million mapped reads, FPKM). (**B** and **C**) Distribution of M5-PETase transcripts, normalised per *recA* gene transcripts, along with depth and latitude and depth and longitude. (**D**) Fraction of samples where expressed M5-PETase genes were detected over the total number of samples for four depth categories (0–100 m, 100–200 m, 200–500 m, > 500 m). (**E**) Mean ±SE M5-PETase transcript abundance, normalised per *recA* gene transcript abundance, at the same four depth layers, including only samples where M5-PETase transcripts were detected.

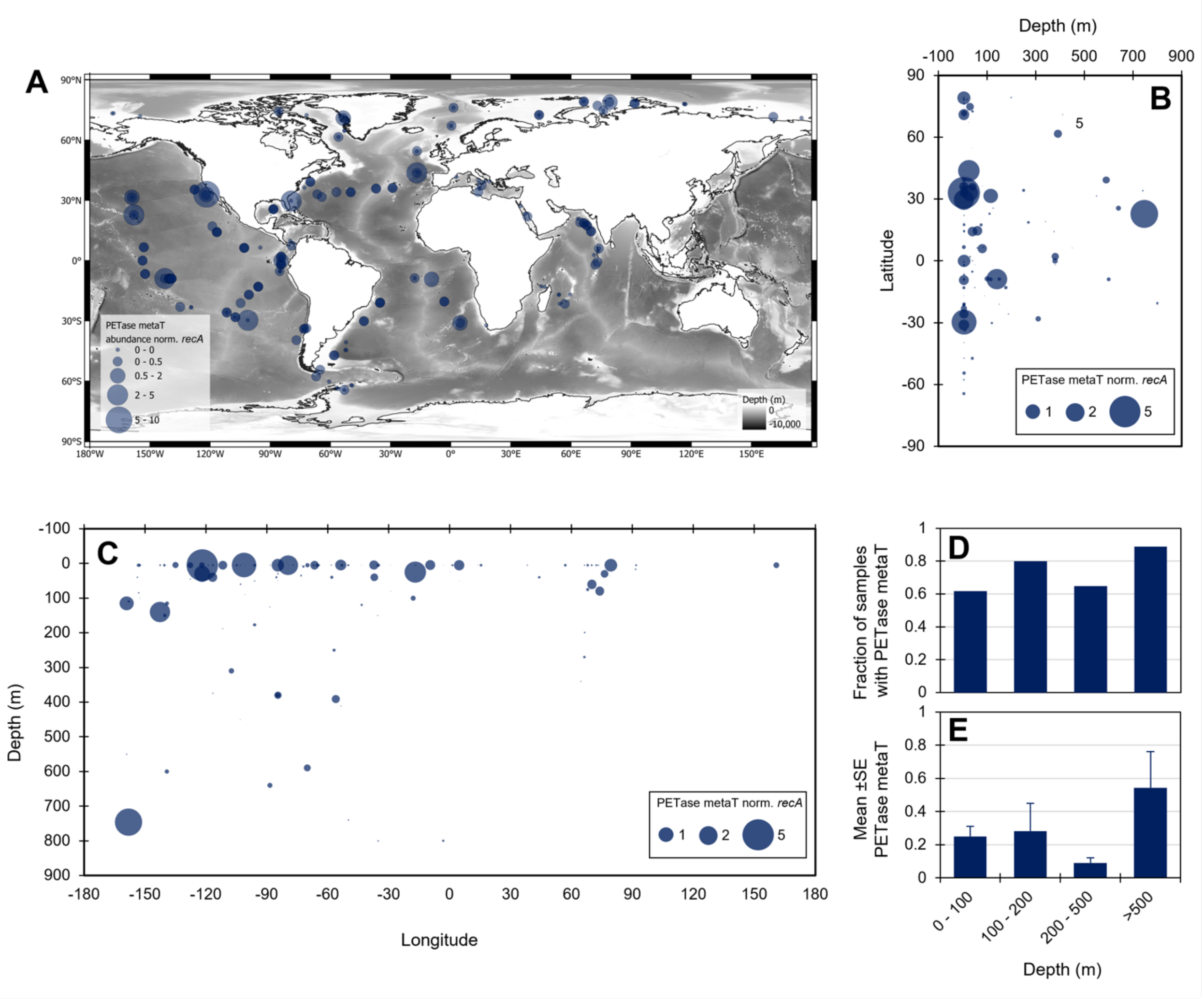

Figure S20. Map of the 506 records of association between plastic samples and M5-PETase transcript samples based on thresholds applied (see **Methods** and **Data S6B**). Plastic samples are represented in red shades per legend, and abundance is expressed as g per km^2. The M5-PETase transcripts are represented in green shades per legend, and abundance is expressed as M5-PETase transcript abundance normalised per *recA* in insert counts. Only M5-PETases associated with plastic sites are represented. These include the samples that fulfil the threshold of i) 5° radius distance from the plastic samples and ii) a depth of < 10m (the maximum detected in this set of samples is 9 m).

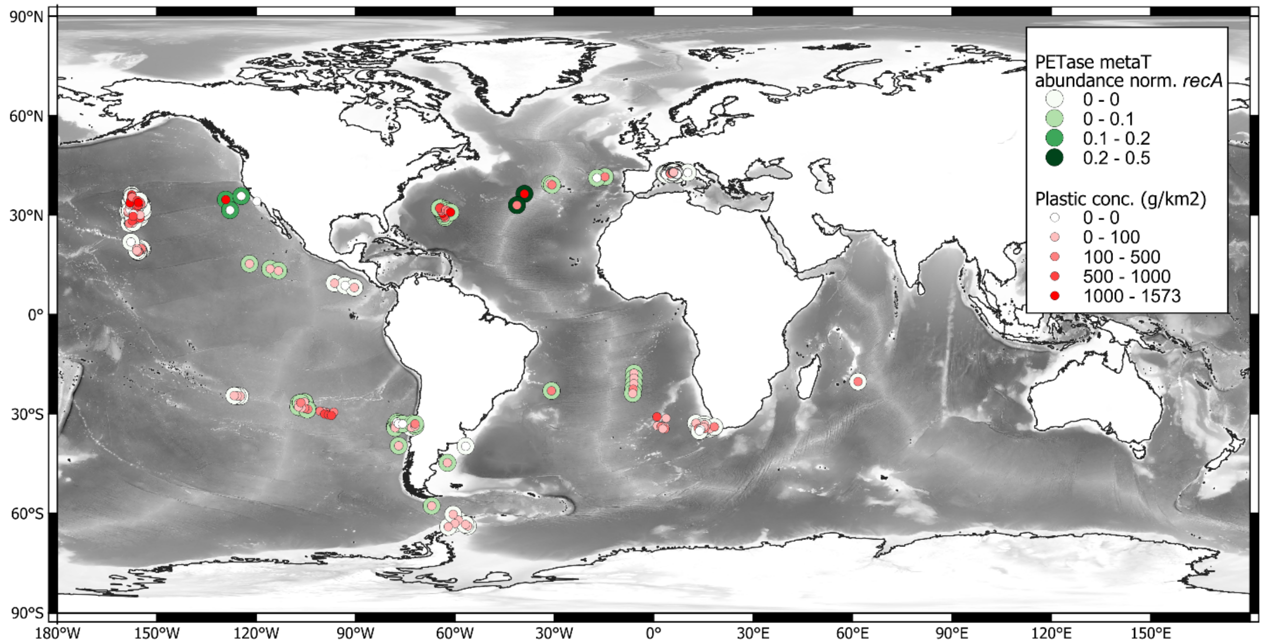

Figure S21. Maximum Likelihood phylogenetic tree showing M5-PETases retrieved from EBI Metagenomic Proteins (MGY), UniProt (sp: swissprot reviewed, tr: unreviewed), reference proteins from NCBI (WP), GenBank metagenomic (GB). Sequences retrieved from terrestrial and water/marine samples are indicated in green and blue, respectively. *Is*PETase was also included as a terrestrial sample.

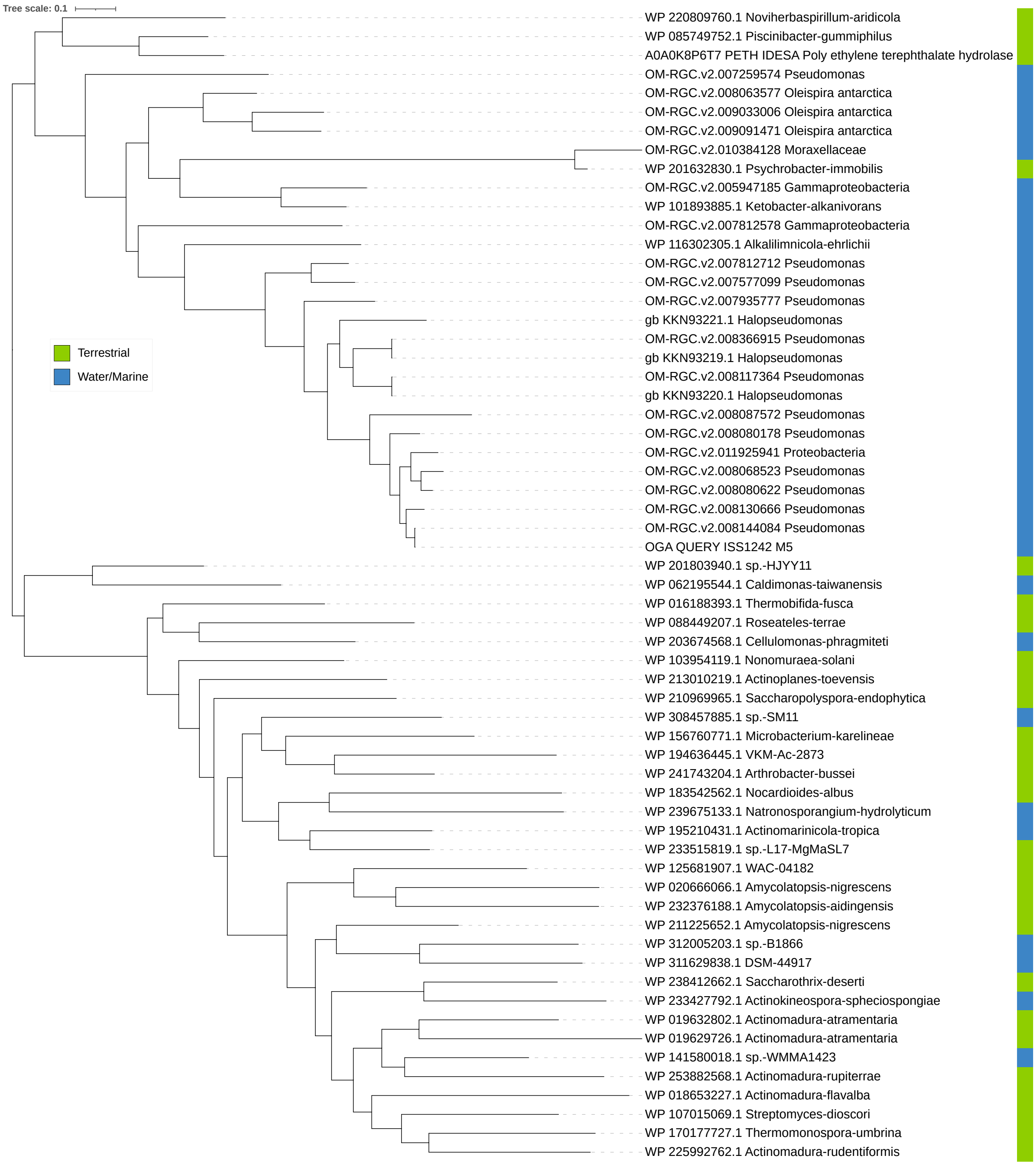

Figure S22. Metagenomic sample in EBI's protein database and taxonomic breakdown of M5-PETase variants across different environmental samples.

0.00 1.00

marine metagenome	wastewater metagenome	metagenome	compost metagenome	sediment metagenome	aquatic metagenome	uncultured Acidobacteriia bacterium	human skin metagenome	human oral metagenome	fermentation metagenome	seawater metagenome	bioreactor metagenome	anaerobic digester metagenome	unknown_metagenome	activated sludge metagenome	freshwater sediment metagenome	biogas fermenter metagenome	rhizosphere metagenome	hot springs metagenome	#	id
6	0	5	0	19	0	0	0	0	0	2	0	0	0	0	0	0	0	0	1	Bacteria;Pseudomonadota;Gammaproteobacteria;Oceanospirillales
21	0	2	1	2	0	0	0	1	0	1	0	0	0	1	0	0	0	0	2	Bacteria;Pseudomonadota;Gammaproteobacteria;Pseudomonadales
1	1	6	1	0	2	2	0	0	0	0	0	0	1	0	2	0	1	1	3	Bacteria;Pseudomonadota;Betaproteobacteria;Burkholderiales
0	0	0	6	0	0	0	0	0	0	0	4	0	4	0	0	0	0	0	4	Bacteria;Actinomycetota;Actinomycetes;Streptosporangiales
5	7	0	0	0	0	0	0	0	0	0	0	0	0	0	0	0	0	0	5	Bacteria;Pseudomonadota;Gammaproteobacteria;Moraxellales
0	0	0	0	1	1	0	0	0	0	0	1	0	0	3	1	0	0	0	6	Bacteria;Myxococcota;Myxococcia;
0	0	0	0	0	0	0	0	0	1	0	1	0	4	0	0	0	0	0	7	Bacteria;Actinomycetota;Actinomycetes;Kitasatosporales
0	0	0	1	0	0	0	0	0	0	0	1	2	0	0	0	0	0	0	8	Bacteria;;;
2	0	0	0	0	0	0	0	0	0	1	0	0	0	0	0	0	0	0	9	Bacteria;Pseudomonadota;Gammaproteobacteria;Arenicellales
1	0	1	0	0	0	0	0	0	0	0	0	0	0	0	0	0	0	0	10	Bacteria;Actinomycetota;Nitriliruptoria;Euzebyales
1	0	0	0	0	0	0	0	0	0	0	0	0	0	0	0	1	0	0	11	Bacteria;Actinomycetota;Actinomycetes;Micrococcales
1	0	1	0	0	0	0	0	0	0	0	0	0	0	0	0	0	0	0	12	Bacteria;Pseudomonadota;Gammaproteobacteria;
0	0	0	0	1	0	0	0	0	0	1	0	0	0	0	0	0	0	0	13	Bacteria;Pseudomonadota;Gammaproteobacteria;Cellvibrionales
2	0	0	0	0	0	0	0	0	0	0	0	0	0	0	0	0	0	0	14	Bacteria;Actinomycetota;Actinomycetes;Micromonosporales
0	0	0	0	0	0	0	0	0	0	0	0	0	1	0	0	0	0	0	15	Bacteria;Actinomycetota;;
0	0	0	0	0	0	0	0	0	0	0	0	0	0	1	0	0	0	0	16	Bacteria;;Deltaproteobacteria;
0	0	0	0	0	0	0	1	0	0	0	0	0	0	0	0	0	0	0	17	Bacteria;Actinomycetota;Actinomycetes;Geodermatophilales
0	0	0	0	0	0	0	0	0	0	0	1	0	0	0	0	0	0	0	18	Bacteria;Pseudomonadota;Gammaproteobacteria;Steroidobacterales
0	0	0	0	0	0	0	0	0	0	0	0	0	1	0	0	0	0	0	19	Bacteria;Actinomycetota;Actinomycetes;Pseudonocardiales
1	0	0	0	0	0	0	0	0	0	0	0	0	0	0	0	0	0	0	20	Bacteria;Actinomycetota;Acidimicrobiia;Acidimicrobiales

Supplementary Tables

Table S1. Established a theoretical "efficiency" score based on the presence of residue substitutions that were previously identified as enhancing PETase activity in engineered laboratory variants of the *Is*PETase. Sources are reported in the reference list.

**Mutation**	**Effect on PETase**	**Score**	**Source**
Y87A	Decreased activity	-1	Han et al., [2]
T88A	Decreased activity	-1	
W159[H/A]	Decreased activity	-1	
S160A *	Decreased activity	+-2	
M161A	Decreased activity	-1	
W185A	Decreased activity	-1	
C203S *	Decreased activity/thermostability	+-2	
I208A	Decreased activity	-1	
S214H	Decreased activity	-1	
C239S *	Decreased activity/thermostability	+-2	
Y87A	Decreased activity	-1	Joo et al. [3]
W159[H/A]	Decreased activity	-1	
S160A *	Decreased activity	+-2	
M161A	Decreased activity	-1	
W185A	Decreased activity	-1	
C203A *	Decreased activity/thermostability	+-2	
D206A *	Decreased activity	+-2	
I208A	Decreased activity	-1	
H237A *	Decreased activity	+-2	
S238F	Decreased activity	-1	
C239A *	Decreased activity/thermostability	+-2	
N241A	Decreased activity	-1	
R280A	Increased activity	1	
R90A	Increased activity	1	Ma et al. [4]
L117F	Increased activity	1	
I208F	Increased activity	1	
R280A	Increased activity	1	Son et al. [5]
W159H + S238F	Increased activity	1	Austin et al. [6]
L117F	Increased activity	1	Cui et al. [7]
R280A	Increased activity	1	
T88M	Increased thermostability	1	Tournier et al. [8]
Q119G	Increase thermostability	1	
S238[I/W]	Increased activity	1	
N241[D/M]	Increased thermostability	1	
N233C + S282C	Increased thermostability	1	

Table S2. Signal peptide prediction generated using the SignalP 6.0 web server based on protein language models (LMs) conducted on the sequences selected for the *in vitro* functional assay and all M5-PETase variants (n=23) detected from ocean metagenomes. The presence of secretion signal peptide is reported for Sec substrates cleaved by SPase I (Sec/SPI) or SPase II (Sec/SPII). Information regarding the cleavage site position and presence probability is also included. A full list of 75 PETase variants possessing the DLH domain and encompassing the M1-M5 motif is reported in **Data S1**. Proteins tested in functional assay *in vitro* are reported in bold.

PETase sequence	Signal Peptide	Cleavage site position	Probability	Functional test
***Is*-WT**	**Sec/SPI**	**between pos. 26 and 27**	**0.947945**	**Yes**
***Is*-S160A**	**Sec/SPI**	**between pos. 26 and 27**	**0.947945**	**Yes**
01.M5	Sec/SPI	between pos. 22 and 23	0.976179	No
02.M5	Sec/SPI	between pos. 25 and 26	0.969832	No
03.M5	Sec/SPI	between pos. 26 and 27	0.848572	No
04.M5	Sec/SPI	between pos. 25 and 26	0.970402	No
05.M5	Sec/SPI	between pos. 25 and 26	0.970402	No
06.M5	Sec/SPI	between pos. 25 and 26	0.970402	No
07.M5	Sec/SPI	between pos. 41 and 42	0.816338	No
08.M5	Sec/SPI	between pos. 25 and 26	0.970402	No
09.M5	Sec/SPI	between pos. 25 and 26	0.969951	No
**10.M5**	**Sec/SPI**	**between pos. 24 and 25**	**0.971847**	**Yes**
11.M5	Sec/SPI	between pos. 25 and 26	0.971780	No
**12.M5**	**Sec/SPI**	**between pos. 22 and 23**	**0.976790**	**Yes**
13.M5	Sec/SPI	between pos. 25 and 26	0.969625	No
14.M5	Sec/SPI	between pos. 25 and 26	0.971780	No
**15.M5**	**Sec/SPI**	**between pos. 25 and 26**	**0.972374**	**Yes**
16.M5	Sec/SPI	between pos. 25 and 26	0.969633	No
17.M5	Sec/SPI	between pos. 25 and 26	0.972055	No
19.M5	Sec/SPI	between pos. 20 and 21	0.995267	No
20.M5	Sec/SPI	between pos. 20 and 21	0.995373	No
21.M5	Sec/SPI	between pos. 20 and 21	0.995594	No
22.M5	Sec/SPII	between pos. 20 and 21	0.995260	No
23.M5	Sec/SPII	between pos. 20 and 21	0.995260	No
24.M5	None*			No
**27.M4**	**Sec/SPI**	**between pos. 24 and 25**	**0.973071**	**Yes**
**36.M4**	**None**			**Yes**
**59.M3**	**None**			**Yes**
**68.M3**	**None**			**Yes**
**70.M2**	**Sec/SPI**	**between pos. 24 and 25**	**0.971847**	**Yes**
**71.M2**	**Sec/SPII**	**between pos. 19 and 20**	**0.995434**	**Yes**
**72.M1**	**None**			**Yes**
**73.M1**	**None**			**Yes**

*Sequence misses the initial portion where the signal peptide is generally present

Table S3. *Pseudomonas* strains (recently reclassified as *Halopseudomonas*) from the marine culture collection of heterotrophic bacteria (MARINHET) obtained from samples collected from *Tara* Oceans (*Tara*) and Malaspina expeditions that were positive for the PETase gene following the PCR-based screening. Identification number (ID) of each isolate, cruise, oceanic location, depth, best hit of the closest cultured match (CCM), and best hit to the closest environmental match (CEM) against the RDP database, with their respective accession numbers and percentage of identity. All strains were cultured on Marine Agar and incubated at room temperature. The strains used for *in vivo* tests are in bold.

ID	Cruise	Location	Depth (m)	Closest Cultured Match	Acc. N.	% Identity
ISS-488	*Tara*	North Atlantic	5	*Pseudomonas* sp.	KJ702653	100
ISS-522	*Tara*	North Atlantic	5	*Pseudomonas pachastrellae*	KM037738	100
**ISS-721**	**Malaspina**	**South Atlantic**	**4003**	***Pseudomonas pachastrellae***	**KJ524490**	**100**
**ISS-1225**	***Tara***	**North Atlantic**	**5**	***Pseudomonas* sp.**	**KJ702653**	**100**
**ISS-1242**	***Tara***	**North Atlantic**	**5**	***Pseudomonas* sp.**	**KJ702653**	**99.81**
ISS-1269	*Tara*	North Atlantic	5	*Pseudomonas pachastrellae*	KM037738	100

Table S4. MHETases from *Pseudomonas* in Ocean samples.

1	id	#members	biome2	prj	run	mg.tax.name	ko-ids	superkingdom	phylum	family	genus	species
2	EarthMicrobiomeGC_0476510883	1	Pelagic	PRJEB4419	ERR599361	marine metagenome	K21105	Bacteria	Pseudomonadota	Pseudomonadaceae	Pseudomonas	-
3	EarthMicrobiomeGC_0372411275	1	Pelagic	PRJEB4352	ERR868420	marine metagenome	K21105	Bacteria	Pseudomonadota	Pseudomonadaceae	Pseudomonas	-
4	EarthMicrobiomeGC_0424816396	2	Pelagic	PRJEB4352	ERR1726724	marine metagenome	K21105	Bacteria	Pseudomonadota	Pseudomonadaceae	Stutzerimonas	Stutzerimonas stutzeri
5	EarthMicrobiomeGC_0322138098	9	Pelagic	PRJEB1788	ERR594322	marine metagenome	K21105	Bacteria	Pseudomonadota	Pseudomonadaceae	Stutzerimonas	Stutzerimonas stutzeri
6	EarthMicrobiomeGC_0381925922	1	Pelagic	PRJEB4352	ERR868467	marine metagenome	K21105	Bacteria	Pseudomonadota	Pseudomonadaceae	Stutzerimonas	Stutzerimonas stutzeri
7	EarthMicrobiomeGC_0307282271	1	Pelagic	PRJEB1787	ERR599159	marine metagenome	K21105	Bacteria	Pseudomonadota	Pseudomonadaceae	Pseudomonas	-
8	EarthMicrobiomeGC_0422810280	2	Pelagic	PRJEB4352	ERR1726714	marine metagenome	K21105	Bacteria	Pseudomonadota	Pseudomonadaceae	Pseudomonas	-
9	EarthMicrobiomeGC_0475882371	2	Pelagic	PRJEB4419	ERR599357	marine metagenome	K21105	Bacteria	Pseudomonadota	Pseudomonadaceae	Pseudomonas	-
10	EarthMicrobiomeGC_0492751926	1	Pelagic	PRJNA257723	SRR5010551	marine metagenome	K21105	Bacteria	Pseudomonadota	Pseudomonadaceae	Stutzerimonas	Stutzerimonas stutzeri
11	EarthMicrobiomeGC_0475291440	1	Pelagic	PRJEB4419	ERR599352	marine metagenome	K21105	Bacteria	Pseudomonadota	Pseudomonadaceae	Pseudomonas	-
12	EarthMicrobiomeGC_0811817306	3	Pelagic	PRJEB14197	ERR2497739	aquatic metagenome	K21105	Bacteria	Pseudomonadota	Pseudomonadaceae	Pseudomonas	-
13	EarthMicrobiomeGC_0023033216	1	Pelagic	PRJEB11402	ERR1078377	metagenome	K21105	Bacteria	Pseudomonadota	Pseudomonadaceae	-	-
14	EarthMicrobiomeGC_0372411276	1	Pelagic	PRJEB4352	ERR868420	marine metagenome	K21105	Bacteria	Pseudomonadota	Pseudomonadaceae	Stutzerimonas	Stutzerimonas stutzeri
15	EarthMicrobiomeGC_0477276134	3	Pelagic	PRJEB4419	ERR599366	marine metagenome	K21105	Bacteria	Pseudomonadota	Pseudomonadaceae	Pseudomonas	-
16	EarthMicrobiomeGC_0811643445	2	Pelagic	PRJEB14197	ERR2497732	aquatic metagenome	K21105	Bacteria	Pseudomonadota	Pseudomonadaceae	Pseudomonas	-
17	EarthMicrobiomeGC_0141535782	2	Pelagic	PRJEB22097	ERR2092774	metagenome	K21105	Bacteria	Pseudomonadota	Pseudomonadaceae	Stutzerimonas	Stutzerimonas chloritidismutans
18	EarthMicrobiomeGC_0811870507	3	Pelagic	PRJEB14197	ERR2497741	aquatic metagenome	K21105	Bacteria	Pseudomonadota	Pseudomonadaceae	Pseudomonas	-
19	EarthMicrobiomeGC_0813873627	1	Pelagic	PRJEB22997	ERR2206760	aquatic metagenome	K21105	Bacteria	Pseudomonadota	Pseudomonadaceae	Stutzerimonas	Stutzerimonas stutzeri
20	EarthMicrobiomeGC_0368715434	1	Pelagic	PRJEB4352	ERR868409	marine metagenome	K21105	Bacteria	Pseudomonadota	Pseudomonadaceae	Stutzerimonas	Stutzerimonas stutzeri
21	EarthMicrobiomeGC_0325837579	1	Pelagic	PRJEB1788	ERR594333	marine metagenome	K21105	Bacteria	Pseudomonadota	Pseudomonadaceae	Pseudomonas	-
22	EarthMicrobiomeGC_0501716992	2	Pelagic	PRJNA318731	SRR3646363	marine metagenome	K21105	Bacteria	Pseudomonadota	Pseudomonadaceae	Stutzerimonas	Stutzerimonas stutzeri
23	EarthMicrobiomeGC_0023393857	7	Pelagic	PRJEB11402	ERR1078379	metagenome	K21105	Bacteria	Pseudomonadota	Pseudomonadaceae	Pseudomonas	-
24	EarthMicrobiomeGC_0023074214	1	Pelagic	PRJEB11402	ERR1078377	metagenome	K21105	Bacteria	Pseudomonadota	Pseudomonadaceae	Pseudomonas	-
25	EarthMicrobiomeGC_0397489605	15	Pelagic	PRJEB4352	ERR1726555	marine metagenome	K21105	Bacteria	Pseudomonadota	Pseudomonadaceae	Pseudomonas	-
26	EarthMicrobiomeGC_0467097941	2	Pelagic	PRJEB4419	ERR594366	marine metagenome	K21105	Bacteria	Pseudomonadota	Pseudomonadaceae	Pseudomonas	-
27	EarthMicrobiomeGC_0395222541	1	Pelagic	PRJEB4352	ERR1726542	marine metagenome	K21105	Bacteria	Pseudomonadota	Pseudomonadaceae	Pseudomonas	-
28	EarthMicrobiomeGC_0318401512	7	Pelagic	PRJEB1788	ERR594309	marine metagenome	K21105	Bacteria	Pseudomonadota	Pseudomonadaceae	Pseudomonas	-
29	EarthMicrobiomeGC_0492283781	6	Pelagic	PRJNA257723	SRR3405540	marine metagenome	K21105	Bacteria	Pseudomonadota	Pseudomonadaceae	Stutzerimonas	Stutzerimonas stutzeri
30	EarthMicrobiomeGC_0811787466	1	Pelagic	PRJEB14197	ERR2497738	aquatic metagenome	K21105	Bacteria	Pseudomonadota	Pseudomonadaceae	Pseudomonas	-
31	EarthMicrobiomeGC_0257711108	1	Pelagic	PRJEB1787	ERR598958	marine metagenome	K21105	Bacteria	Pseudomonadota	Pseudomonadaceae	Pseudomonas	-
32	EarthMicrobiomeGC_0317637999	1	Pelagic	PRJEB1788	ERR594305	marine metagenome	K21105	Bacteria	Pseudomonadota	Pseudomonadaceae	Stutzerimonas	Stutzerimonas stutzeri
33	EarthMicrobiomeGC_0389886746	2	Pelagic	PRJEB4352	ERR868507	marine metagenome	K21105	Bacteria	Pseudomonadota	Pseudomonadaceae	Stutzerimonas	Stutzerimonas stutzeri
34	EarthMicrobiomeGC_0471577919	1	Pelagic	PRJEB4419	ERR594407	marine metagenome	K21105	Bacteria	Pseudomonadota	Pseudomonadaceae	Pseudomonas	-
35	EarthMicrobiomeGC_0421909282	4	Pelagic	PRJEB4352	ERR1726708	marine metagenome	K21105	Bacteria	Pseudomonadota	Pseudomonadaceae	Pseudomonas	-
36	EarthMicrobiomeGC_0367051472	1	Pelagic	PRJEB4352	ERR868399	marine metagenome	K21105	Bacteria	Pseudomonadota	Pseudomonadaceae	Pseudomonas	-
37	EarthMicrobiomeGC_0334771189	2	Pelagic	PRJEB4352	ERR538188	marine metagenome	K21105	Bacteria	Pseudomonadota	Pseudomonadaceae	Pseudomonas	-
38	EarthMicrobiomeGC_0418372728	2	Pelagic	PRJEB4352	ERR1726685	marine metagenome	K21105	Bacteria	Pseudomonadota	Pseudomonadaceae	Pseudomonas	-
39	EarthMicrobiomeGC_0467438529	7	Pelagic	PRJEB4419	ERR594371	marine metagenome	K21105	Bacteria	Pseudomonadota	Pseudomonadaceae	Stutzerimonas	Stutzerimonas stutzeri

References

1. Bollinger A et al. A novel polyester hydrolase from the marine bacterium *Pseudomonas aestusnigri* – Structural and functional insights. *Front Microbiol* 2020;**11**. https://doi.org/10.3389/fmicb.2020.00114
2. Sanz-Sáez I et al. Diversity and distribution of marine heterotrophic bacteria from a large culture collection. *BMC Microbiol* 2020;**20**:207. https://doi.org/10.1186/s12866-020-01884-7
3. Burbick CR et al. An update on novel taxa and revised taxonomic status of bacteria (including members of the phylum Planctomycetota) isolated from aquatic host species described in 2018 to 2021. *J Clin Microbiol* 2023;**61**. https://doi.org/10.1128/jcm.01426-22
4. Rudra B, Gupta RS. Phylogenomic and comparative genomic analyses of species of the family Pseudomonadaceae: Proposals for the genera Halopseudomonas gen. nov. and Atopomonas gen. nov., merger of the genus Oblitimonas with the genus Thiopseudomonas, and transfer of some misc. *Int J Syst Evol Microbiol* 2021;**71**. https://doi.org/10.1099/ijsem.0.005011
5. Romanenko LA et al. Pseudomonas pachastrellae sp. nov., isolated from a marine sponge. *Int J Syst Evol Microbiol* 2005;**55**:919–924. https://doi.org/10.1099/ijs.0.63176-0
6. Olm MR et al. dRep: a tool for fast and accurate genomic comparisons that enables improved genome recovery from metagenomes through de-replication. *ISME J* 2017;**11**:2864–2868. https://doi.org/10.1038/ismej.2017.126
7. Waterhouse A et al. SWISS-MODEL: homology modelling of protein structures and complexes. *Nucleic Acids Res* 2018;**46**:W296–W303. https://doi.org/10.1093/nar/gky427
8. Han X et al. Structural insight into catalytic mechanism of PET hydrolase. *Nat Commun* 2017;**8**:2106. https://doi.org/10.1038/s41467-017-02255-z
9. Joo S et al. Structural insight into molecular mechanism of poly(ethylene terephthalate) degradation. *Nat Commun* 2018;**9**:382. https://doi.org/10.1038/s41467-018-02881-1
10. Ma Y et al. Enhanced poly(ethylene terephthalate) hydrolase activity by protein engineering. *Engineering* 2018;**4**:888–893. https://doi.org/10.1016/j.eng.2018.09.007
